# Oxyberberine alleviates lipopolysaccharide-induced intestinal barrier disruption and inflammation in human colonic Caco-2 cells *in vitro*

**DOI:** 10.3389/fphar.2024.1496874

**Published:** 2025-01-07

**Authors:** Cailan Li, Jiahao Wang, Hongmei Yang, Shuang Luo, Qiang Lu

**Affiliations:** ^1^ Department of Pharmacology, Zunyi Medical University, Zhuhai, China; ^2^ Key Laboratory of Basic Pharmacology of Ministry of Education and Joint International Research Laboratory of Ethnomedicine of Ministry of Education, Zunyi Medical University, Zunyi, China; ^3^ Key Laboratory of Basic Pharmacology of Guizhou Province and School of Pharmacy, Zunyi Medical University, Zunyi, China; ^4^ Faculty of Health Sciences, University of Macau, Taipa, Macao SAR, China; ^5^ Shenzhen Traditional Chinese Medicine Hospital, The Fourth Clinical Medical College of Guangzhou University of Chinese Medicine, Shenzhen, China; ^6^ Department of Pharmaceutical Sciences, Zunyi Medical University, Zhuhai, China

**Keywords:** ulcerative colitis, oxyberberine, intestinal barrier, inflammation, Nrf2, NF-κB

## Abstract

**Background:**

Oxyberberine (OBB) is a naturally occurring isoquinoline alkaloid that is believed to possess various health-promoting properties, including anti-fungus, hepatoprotection, anti-inflammation, and anti-intestinal mucositis effects. Despite several studies reporting the health benefits of OBB in treating ulcerative colitis (UC), its specific mechanism of action has yet to be fully elucidated.

**Purpose:**

This investigation is designed to explore the potential protective efficacy of OBB and the latent mechanism using an *in vitro* model of UC-like inflammatory intestinal cells.

**Methods:**

Caco-2 cells were pretreated with OBB and subsequently exposed to lipopolysaccharide (LPS). The transepithelial electrical resistance (TEER), paracellular permeability, and the distribution and expression of tight- and adherent junction proteins were determined to assess barrier integrity. The levels of proinflammatory cytokines, reactive oxygen species (ROS), Nrf2, and NF-κB signaling cascade were analyzed via ELISA, qRT-PCR, immunofluorescence, or Western blotting.

**Results:**

OBB was found to mitigate the effects of LPS on Caco-2 cell monolayers, as evidenced by the improvement in TEER and the decrease in FITC-dextran flux. Moreover, OBB ameliorated the LPS-induced decrease in the distribution and expression of several tight junction markers, including ZO-1, occludin, and E-cadherin. In addition, OBB treatment effectively inhibited LPS-induced increases in ROS, apoptosis, and Keap1 and decreases in Nrf2 and HO-1. LPS-induced elevations in nuclear NF-κB p65 and p-IκBα were suppressed by OBB. In addition, ML385, an antagonist of Nrf2, abolished the protective role of OBB.

**Conclusion:**

OBB has a pronounced beneficial effect on LPS-induced damage to enteral barrier function, and the regulation of the Nrf2/NF-κB pathway is an important mechanism responsible for the protection afforded by OBB.

## 1 Introduction

Ulcerative colitis (UC), a main subtype of inflammatory bowel disease, is a chronic aspecific inflammation illness that primarily influences the distal colon and rectum (Feuerstein et al., 2019). At present, the occurrence and morbidity of UC are on the rise around the world, particularly in less-developed nations (Wei et al., 2021). Classical characteristics of UC involve bloody diarrhea, bellyache, urgent stools, and weight reduction. Clinically, patients suffering from UC often relapse and are susceptible to developing UC-associated cancer (Follin-Arbelet et al., 2023). To date, the therapeutic drugs of UC include aminosalicylic acids, thiopurines, immunosuppressors, glucocorticoids, and biologics (Li et al., 2022). However, the long-term use of these medicines has poor effectiveness, frequently results in serious adverse reactions, and imposes a weighty burden on society and families (Nasir et al., 2022; Tsujii et al., 2022).

A complicated interaction of inheritance, immunization, environment, and intestinal flora is considered to be the cause of UC (Lu et al., 2023; Ning et al., 2024). The exact etiology and pathogenic mechanism are still unclear. In recent years, numerous studies have proved that impaired gut barrier function is one of the primary traits of UC, and the impairment of enterocytes exerts an important effect on the pathogenesis of UC (Seidelin et al., 2021; Tian and Zhang, 2022). The gut epithelial barrier is a mechanical and physiological barrier that blocks the entry of antigens and pathogens into the bloodstream. It has been reported that disorders of the enteral barrier function could exacerbate inflammation and lead to the occurrence and development of UC (Han et al., 2022). However, no FDA-approved therapies target the epithelial barrier at present. Therefore, the search for efficient medicines that can reinforce the gut epithelial barrier is of enormous importance for the management of UC (Li C. L. et al., 2021; Zhang et al., 2023).

Lipopolysaccharide (LPS) exerts an important effect on enteric and full-body inflammation responses. Numerous studies have proved that LPS enhances tight junction (TJ) penetration and disrupts gut barrier functions (Han et al., 2023; Saxami et al., 2021). Moreover, continuous inflammatory stimulation leads to impaired gut TJ barrier function, which generates more pro-inflammation factors, creating a vicious spiral (Li M. H. et al., 2024). Thus, strategies that reduce inflammation reactions may be exciting therapeutic options for UC. A few *in vitro* experiments have proved that natural products such as berberine could alleviate LPS-elicited gut barrier impairment (Zhang et al., 2020). A ginger extract exhibited protection on the gut epithelium, with transepithelial electrical resistance (TEER) values rising compared to LPS-treated Caco-2 cells (Kim et al., 2017). Probiotic formulations prevented enteral barrier damage by modulating the expression of TJ proteins and inhibiting inflammatory responses (di Vito et al., 2022).

Berberine is a yellow coloring alkaloid acquired from the rhizome of *Coptidis chinensis* Franch. Over the years, berberine has attracted wide attention for its extensive pharmacological action comprising anti-tumor, anti-inflammation, anti-oxidation, and anti-bacterial activity (Khoshandam et al., 2022). A few studies showed that the application of berberine was impeded by its inferior bioavailability and rapid systemic elimination. Even with high dosages of berberine, the berberine concentrations are very low in serums and tissues (Thomas et al., 2021). Multiple studies have indicated that the metabolic products of berberine may contribute to its pharmacological actions *in vivo* (Cheng et al., 2022; Wang et al., 2017).

**FIGURE 1:**
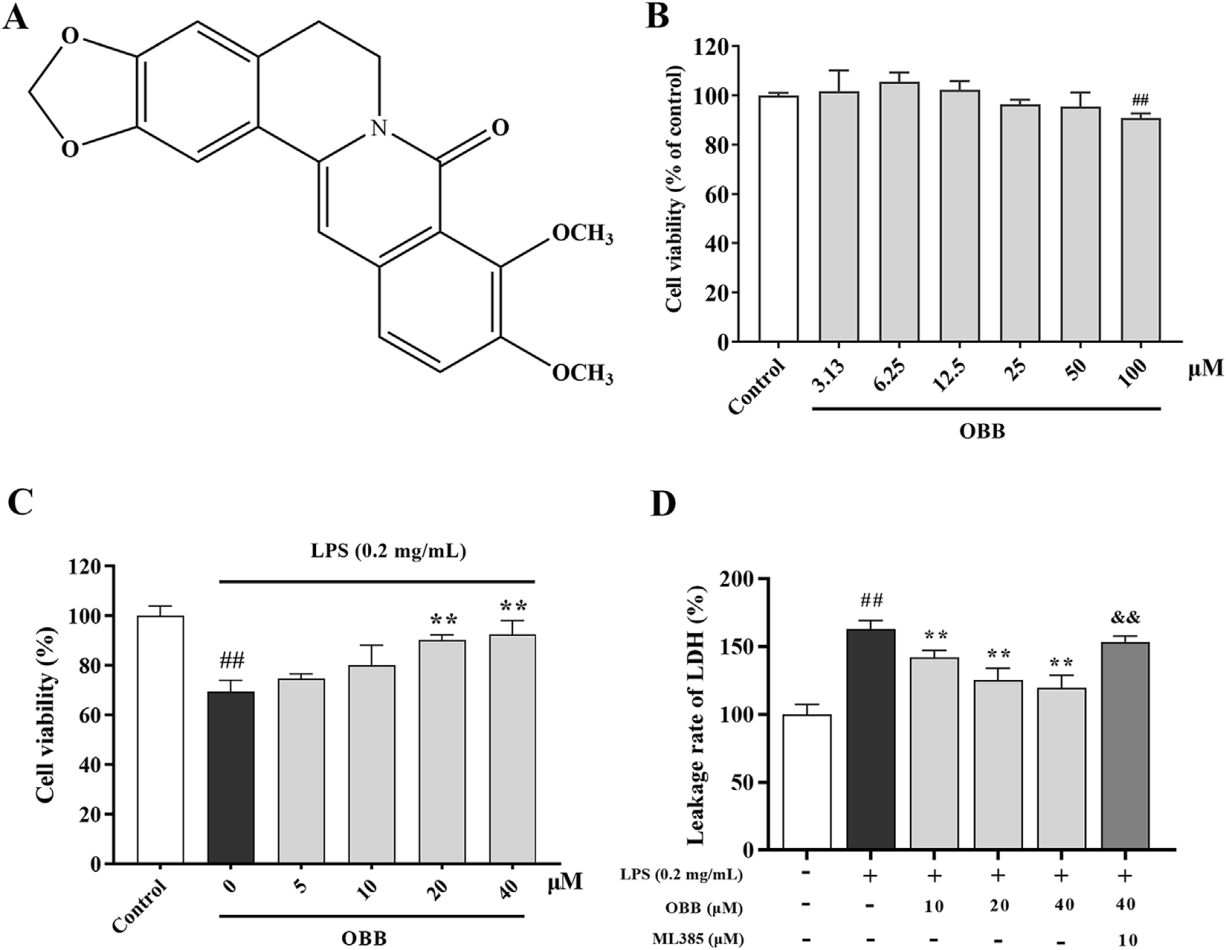
Effects of OBB on Caco-2 cell viability and cytotoxicity. **(A)** Chemical composition of OBB. The Caco-2 viability was determined at increasing concentrations of OBB in the absence **(B)** or presence **(C)** of LPS (0.2 mg/mL) for 24 h. Cell viability was estimated using a CCK-8 assay. **(D)** Cytotoxicity was determined by LDH release. Results are expressed as mean ± SD (n = 6). ^##^ *P* < 0.01 vs. Control; **P < 0.01 vs. LPS group; ^&&^ *P* < 0.01 vs. OBB (40 μM) group.

In our previous research, we first discovered that oxyberberine (OBB, C_20_H_17_NO_5_, Figure 1A), a gut microbiota-mediated oxidized product of berberine with a more active lactam ring, had a more favorable safety profile than berberine (Li et al., 2020). *In vivo*, OBB was an absorbable form of berberine administration, with an enteral absorption rate 5-fold higher than berberine (Feng et al., 2015). OBB is an isoquinoline alkaloid acquired from *Coptidis chinensis* Franch., *Phellodendron chinense* Schneid., and *Argemone mexicana* Linn. (Qian et al., 2015; Singh et al., 2016) that has a wide range of health-promoting properties. Importantly, OBB was shown to be more effective than berberine in various biological functions, including anti-inflammation (Li et al., 2019), anti-intestinal mucositis (Huang et al., 2022), anti-fungus (Singh et al., 2009), and anti-hypoglycemia (Dou et al., 2021). In previous studies, we first revealed that OBB alleviated dextran sulfate sodium (DSS) or 2, 4, 6-trinitrobenzenesulfonic acid (TNBS)-elicited murine UC via retarding inflammation, oxidative stress, and modulating the enteral microbiota (Li et al., 2020; Li et al., 2023). However, the specific mechanism is still not well illuminated.

To provide more profound evidence of the anti-UC effect and mechanism of OBB, our team sought to elucidate the likely role and molecular mechanism of OBB using an LPS-induced Caco-2 cell model. As far as we know, the present investigation is the foremost trial investigating the possible protection and underlying mechanisms of OBB in a UC cell model. The results indicated that OBB had a pronounced beneficial effect on LPS-mediated damage to gut barrier function and that regulation of the Nrf2/NF-κB pathway was a pivotal target responsible for the protection afforded by OBB. This research will provide further support for the potential of OBB as an innovative treatment for UC.

## 2 Materials and methods

### 2.1 Chemicals and reagents

OBB was synthesized following the procedure described in our prior research (Li et al., 2020). ML385 was sourced from APExBIO. LPS (from *Escherichia coli* 055:B5) was obtained from Sigma-Aldrich (St. Louis, MO, United States). Human ELISA kits, including TNF-α, IL-1β, IL-2, and IL-6, were supplied by MLBIO (Shanghai, China), and IL-8 and IL-12 kits were supplied by Meimian (Jiangsu, China). The reactive oxygen species (ROS) detection kit was obtained from Meilunbio (Dalian, China). Lactate dehydrogenase (LDH) was obtained from Beyotime Biotechnology. The Annexin V-FITC/PI double staining apoptosis determination kit was acquired by BestBio (Shanghai, China). Primary antibodies for NF-κB p65, ZO-1, occludin, and E-cadherin were provided by Baijia Biosciences (Taizhou, China). Nrf2, Keap1, HO-1, IκBα, and p-IκBα were purchased from Affinity Biosciences (OH, United States). DyLight 488 AffiniPure Goat Anti-Rabbit IgG was obtained from Earthox. DAPI was provided by Solarbio (Beijing, China).

### 2.2 Cell culture

Human colon cancer cell line Caco-2 was purchased from iCell Bioscience Inc. (Shanghai, China). Caco-2 cells were cultivated in MEM media supplemented with 10% fetal bovine serum (FBS) and 1% penicillin-streptomycin antibiotics at 37°C in 5% CO_2_. The medium was replaced every 2 days. Cells were starved in serum-free media for 12 h prior to every test. Cells at passages 10–20 were used in the experiments.

### 2.3 Cell viability experiment

The cellular viability of OBB was assessed via a CCK-8 experiment based on the manufacturer’s specifications. Caco-2 cells (1 × 10^4^/well) were seated in a 96-well microplate and cultured in medium containing 10% FBS for 36 h. Then, the cells were washed twice with PBS and cultured in serum-free medium for 12 h. The cells were then subjected to distinct concentrations of OBB for 24 h or to OBB for 2 h and supplemented with LPS for a further 24 h. Subsequently, 10 μL CCK-8 solution was supplemented and further fostered for 2 h. Finally, the OD values were determined at 450 nm by a microplate reader.

### 2.4 LDH assay

LDH is a stable cytosolic enzyme present throughout the cell. It is promptly secreted into the supernatant of cell cultures when there is membrane damage, which is a critical characteristic of cells experiencing apoptosis, necrosis, and other types of cell injury (Kumar et al., 2018). The cytotoxic effects of OBB (10 μM, 20 μM, 40 μM) or OBB in combination with ML385 on Caco-2 cells were assessed via measurement of LDH leakage into the extracellular medium according to the manufacturer’s instructions.

### 2.5 Determination of TEER and penetrability

TEER was inspected to estimate the barrier integrality of Caco-2 cells. Cells (1 × 10^4^ cells/well) were cultured in the upper chamber of the transwell, complete medium was added to the lower chamber, and the plate was placed in a 37°C, 5% CO_2_ cell culture incubator. Cellular TEER values were detected at fixed times using a Millicell^®^ ERS-2 cytoresistometer (Millipore, Merk, United States) until TEER values stabilized to indicate monolayer epithelial barrier formation. After 14–18 days, the cell resistance value was basically stable, and the cell monolayer barrier had formed. The transwell plates were gently washed twice with PBS, and the TEER values were measured at 0 h, 2 h, 6 h, 12 h, 24 h, and 48 h after adding the drugs.

The penetrability of the Caco-2 single-cell layer was determined through the flux of FITC-dextran (150 kDa, MCE, United States). FITC-dextran solution (1 mg/mL, dissolved in HBSS) was supplemented into the upper chamber of the transwell, and HBSS was added into the lower chamber. After incubating for 1 h, the lower chamber aliquots were gathered for the detection of fluorescence at 490 nm (excited wavelength) and 520 nm (emissive wavelength).

### 2.6 Measurement of cytokine levels

Caco-2 cells (1 × 10^4^ cells/well) were seeded into 96-well plates and cultured in medium containing 10% FBS for 36 h. Then, the cells were washed twice with PBS and cultured in serum-free medium for 12 h. The cells were subsequently pre-treated with different concentrations of OBB (10 μM, 20 μM, and 40 μM) or OBB (40 μM) + ML385 (10 μM) for 2 h, and treatment was followed with LPS (0.2 mg/mL) for 24 h. The concentrations and exposure periods of the compounds used in the study are based on preliminary experiments. The supernatants were obtained, and the levels of inflammatory cytokines (TNF-α, IL-1β, IL-2, IL-6, IL-8, and IL-12) were assessed based on the directions of nitric oxide assay kit and ELISA kits, respectively.

### 2.7 Determination of ROS content

ROS was assessed using a specific probe DCFH-DA, which can rapidly penetrate the cell membrane and be converted into DCFH within the cell. In the presence of ROS, DCFH was oxidized to form the fluorescent green substance DCF (Kim and Xue, 2020). Both a fluorescent microscope and spectrofluorometry were used to estimate ROS levels. Cells were dispersed in distinct concentrations of OBB or OBB combined ML385 for 2 h, followed by adding LPS for further incubation for 24 h. The cells were then washed with pre-cooling PBS and incubated with 10 μM DCFH-DA at 37°C for 20–30 min. Fluorescence intensity was examined at an excitation wavelength of 488 nm and an emission wavelength of 525 nm.

### 2.8 Immunofluorescent experiment

Cells (5 × 10^4^/well) were cultured on a 24-well plate. After preparation, the cells were rinsed using PBS three times and fixed using 4% paraformaldehyde for 30 min. Triton-100 buffer (0.2%) was then applied to penetrate cells at room temperature for 15 min. After being washed with PBS three times, the cells were blocked using 2% BSA for 30 min. Subsequently, the primary antibodies, including ZO-1, occludin, and E-cadherin, were supplemented and coincubated with the cells at 4°C overnight. Then, the cells were coincubated with DyLight 488 AffiniPure Goat Anti-Rabbit IgG for 1 h under ambient temperature. After staining with DAPI solution under dark conditions for 10 min, a fluorescence microscope was employed to inspect and photograph the cells.

### 2.9 Apoptosis analysis

Cells were excited using 0.2 mg/mL LPS for 24 h with or without OBB and OBB combined with ML385 pretreatment for 2 h. Then, cells were assimilated, gathered, and rinsed using pre-cooling PBS. Flow cytometric analysis was conducted based on the instructions of the apoptotic kit.

### 2.10 RT-PCR

Total RNA was acquired with TRIzol reagent following the manufacturer’s specification. cDNA was synthesized from total RNA. The conditions of RT-PCR were 95°C for 30 s, then 95°C for 15 s, and 60°C for 30 s, repeated 40 times. The reaction was carried out by a Real-Time PCR System (Bio-rad, CA, United States). The cycle threshold (Ct) value was counted and normalized to the abundance of a reference gene GAPDH. The specific primers are as follows: COX-2, forward, GGG​TTG​CTG​GTG​GTA​GGA​ATG, reverse, CAT​AAA​GCG​TTT​GCG​GTA​CTC​AT; iNOS, forward, GAC​TCA​CAG​CCT​TTG​GAC​CTC​A, reverse, GCT​GGA​TGT​CGG​ACT​TTG​TAG​ATT, GAPDH, forward, GGA​AGC​TTG​TCA​TCA​ATG​GAA​ATC, reverse, TGA​TGA​CCC​TTT​TGG​CTC​CC.

### 2.11 Western blot analysis

Total, cytoplasmic, and nuclear proteins were abstracted from Caco-2 cells using corresponding reagent kits. Protein levels were then detected by adopting a BCA protein assay kit (Beyotime). The protein sample was segregated using SDS-PAGE and shifted to a PVDF membrane. Following closure with 5% skim milk, the membrane was coincubated with proper primary antibodies overnight at 4°C and subsequently coincubated with secondary antibodies at ambient temperature for 2 h. Following three rinses with TBST, the blots were visualized through electrochemiluminescence plus reagent (Invitrogen). Image J software was used to quantify the band intensity.

### 2.12 Statistical analysis

The results are indicated by the mean ± standard deviation (SD). All statistics were determined using the IBM SPSS26.0 statistical program. All assays were performed at three or more times. One-way ANOVA followed by Tukey’s *post hoc* tests was adopted to compare the discrepancies between each group. The degree of significance was set at *P* < 0.05.

## 3 Results

### 3.1 Cell viability of OBB in Caco-2 cells

The possible cytotoxic impact of OBB (chemical structure shown in Figure 1A) was evaluated by the CCK-8 assay in Caco-2 cells. Figure 1B demonstrated that OBB alone did not display any cytotoxicity compared to the normal control up to 50 μM. In contrast, at a higher concentration of 100 μM, considerable cell toxicity was observed compared to the control group. Moreover, as shown in Figure 1C, 0.2 mg/mL LPS intervention for 24 h reduced the cellular activity compared to the control group (*P* < 0.01). However, OBB (10 μM, 20 μM, and 40 μM) elevated the cellular activity in a dosage-reliant mode compared to LPS alone (*P* < 0.05). Therefore, OBB at the concentrations of 10 μM, 20 μM, and 40 μM were employed in the below studies according to the cytotoxic result.

Nrf2 plays a role as a modulator in inhibiting oxidative damage and inflammation of colitis, and our previous research showed that OBB may reduce DSS-evoked colitis in mice via activating Nrf2. Therefore, ML385, a specific suppressor of Nrf2, was used in this study. LDH leakage was considered a marker of cellular toxicity. We investigated the possibility that the LPS-induced cytotoxic response to LDH leakage was abolished by OBB. As shown in Figure 1D, LPS (0.2 mg/mL) caused a marked cytotoxic influence on LDH leakage. However, pretreatment with OBB (10 μM, 20 μM, and 40 μM) reduced the cytotoxicity elicited by LPS, which was reversed by ML385.

### 3.2 OBB alleviates LPS-induced intestinal epithelial barrier disruption by activating Nrf2 signaling

**FIGURE 2:**
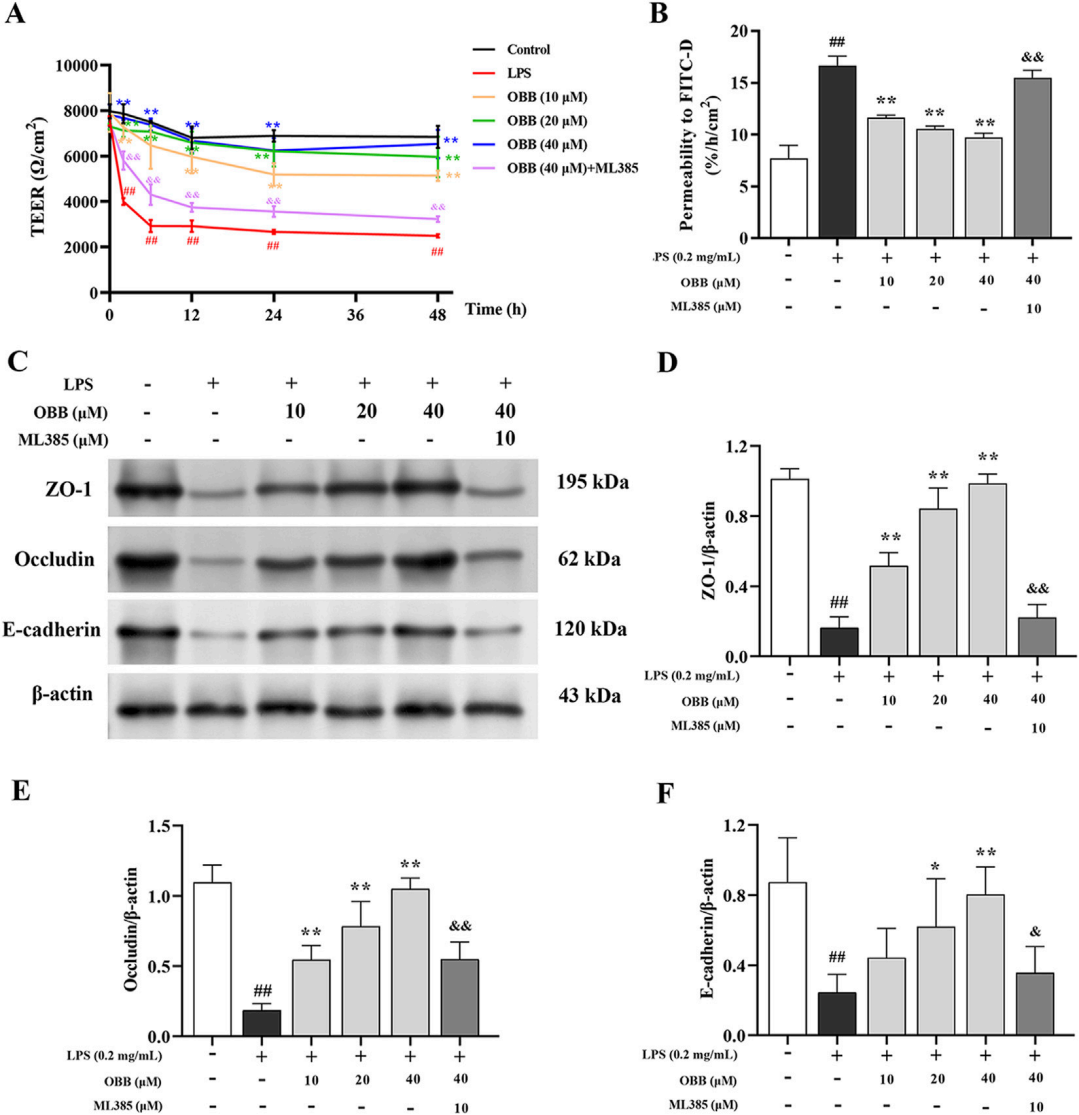
Effect of OBB on the intestinal barrier function in LPS-stimulated Caco-2 cells. **(A)** TEER values were measured at 0 h, 2 h, 6 h, 12 h, 24 h, and 48 h following LPS stimulation with or without OBB and OBB in combination with ML385 treatment. **(B)** The intestinal barrier permeability of Caco-2 cells was determined by employing the FITC-dextran flux assay. **(C–F)** The protein expressions of ZO-1, occludin, and E-cadherin in Caco-2 cells were inspected via Western blotting. β-actin was used as the internal control. Values in the figures are represented by mean ± SD (n = 3). ^##^ *P* < 0.01 vs. Control; *P < 0.05, **P < 0.01 vs. LPS group; ^&^ *P* < 0.05, ^&&^ *P* < 0.01 vs. OBB (40 μM) group.

TEER was employed to detect the single-layer integrity of Caco-2 cells. TEER levels were evaluated at 0 h, 2 h, 6 h, 12 h, 24 h, 36 h, and 48 h following LPS with or without OBB (10 μM, 20 μM, and 40 μM) and OBB combined with ML385 (10 μM) treatment. As shown in Figure 2A, LPS contributed to the marked decline of TEER values following 2 h interventions, which continued up to 48 h after treatment. In contrast, OBB elevated TEER values in a dose-dependent mode, which were then converted by ML385.

In addition, we determined the permeation of large solutes in Caco-2 cells by adopting the FITC-dextran flux test. Compared with the untreated group, the FITC-dextran levels in the basal chamber of the LPS group increased (*P* < 0.05). In contrast, OBB administration dose-dependently decreased the leakage of FITC-dextran caused by LPS treatment compared to the LPS group (Figure 2B), which was then attenuated by ML385. These findings indicated that OBB can attenuate LPS-mediated enteral epithelial barrier impairment *in vitro*.

Modifications in TJ protein expressions have been implicated in gut barrier disturbances triggered by inflammatory factors (Barbara et al., 2021). Therefore, we investigated the function of OBB on the expression of the TJ protein ZO-1, occludin, and E-cadherin in Caco-2 cells exposed to LPS. As illustrated in Figures 2C–F, the protein expression of cellular ZO-1, occludin, and E-cadherin was reduced by the treatment with LPS compared to the blank group (*P* < 0.05). The OBB application restored the levels of ZO-1, occludin, and E-cadherin in a dose-dependent mode (*P* < 0.05 vs. LPS), which was subsequently reversed by ML385.

### 3.3 OBB restrains the morphologic destruction of TJ proteins in LPS-evoked Caco-2 cells

**FIGURE 3:**
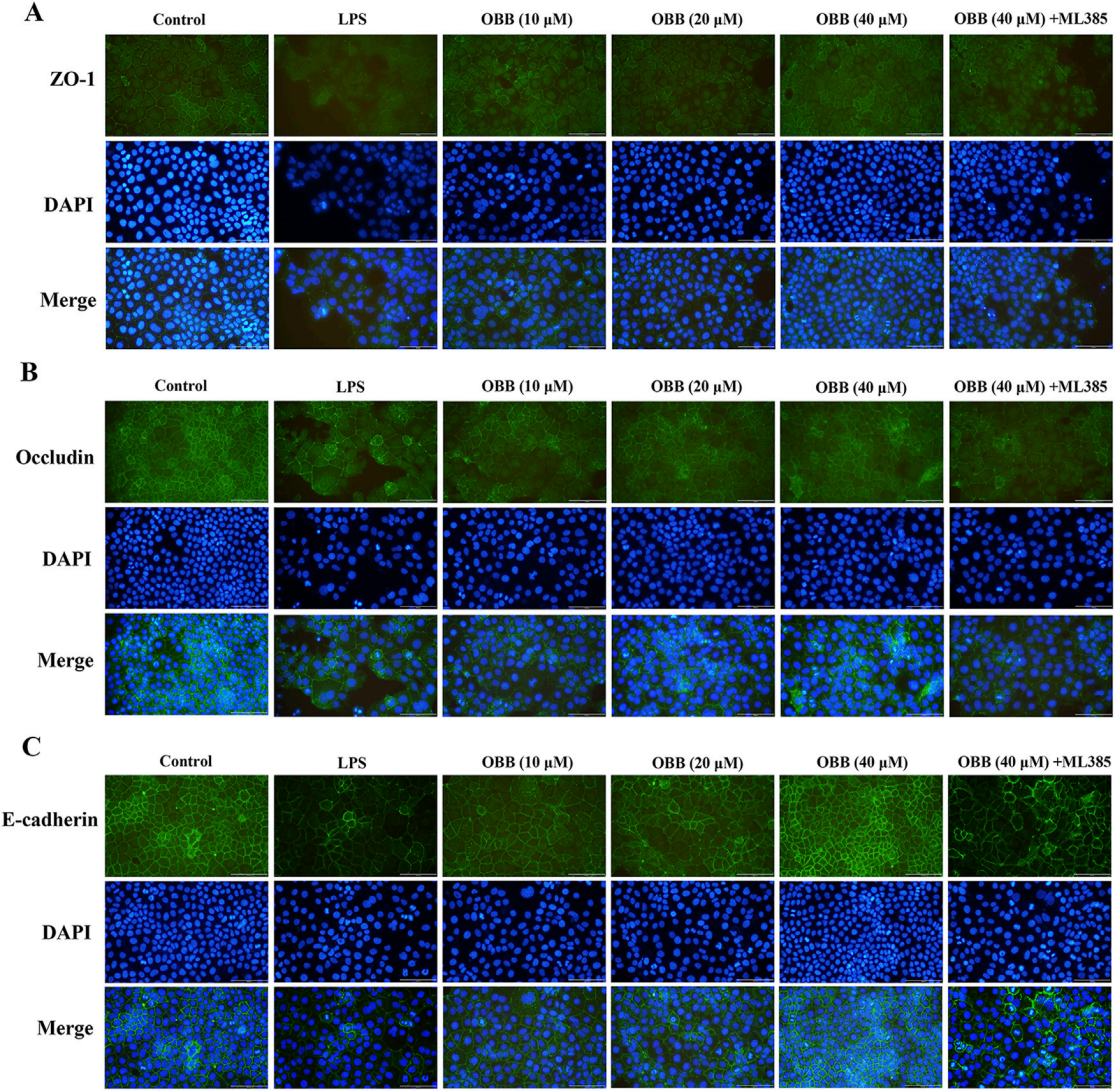
OBB suppresses the morphologic destruction of tight junctions evoked by LPS. Typical tight junction proteins ZO-1 **(A)**, occludin **(B)**, and E-cadherin **(C)** were visualized through immunofluorescent analysis and DAPI staining of nuclei in Caco-2 cells (scale bar 100 μm).

Previous evidence has revealed that inflammation-induced gut barrier dysfunction was involved in the structural destruction and relocalization of TJ proteins (Cao et al., 2013). Therefore, we sought to determine whether OBB altered the morphological localization of TJ proteins in Caco-2 cells following exposure to LPS through immunofluorescent analysis. As shown in Figure 3, in the control group, the TJ proteins ZO-1, occludin, and E-cadherin were each localized to the intercellular TJ alongside the cell edge. Caco-2 cells treated with LPS exhibited a distinct reorganization of the TJ proteins ZO-1, occludin, and E-cadherin, resulting in irregular and discontinuous dispersion patterns. However, OBB therapy significantly mitigated the LPS-induced morphologic destruction of ZO-1, occludin, and E-cadherin in Caco-2 cells, and this protective effect was then mitigated by ML385. These results showed that OBB maintained the integrality of Caco-2 cells by restraining the decline and reconstitution of TJ proteins in LPS-evoked Caco-2 cells.

### 3.4 Effects of OBB on LPS-elicited cellular inflammation in Caco-2 cells

**FIGURE 4:**
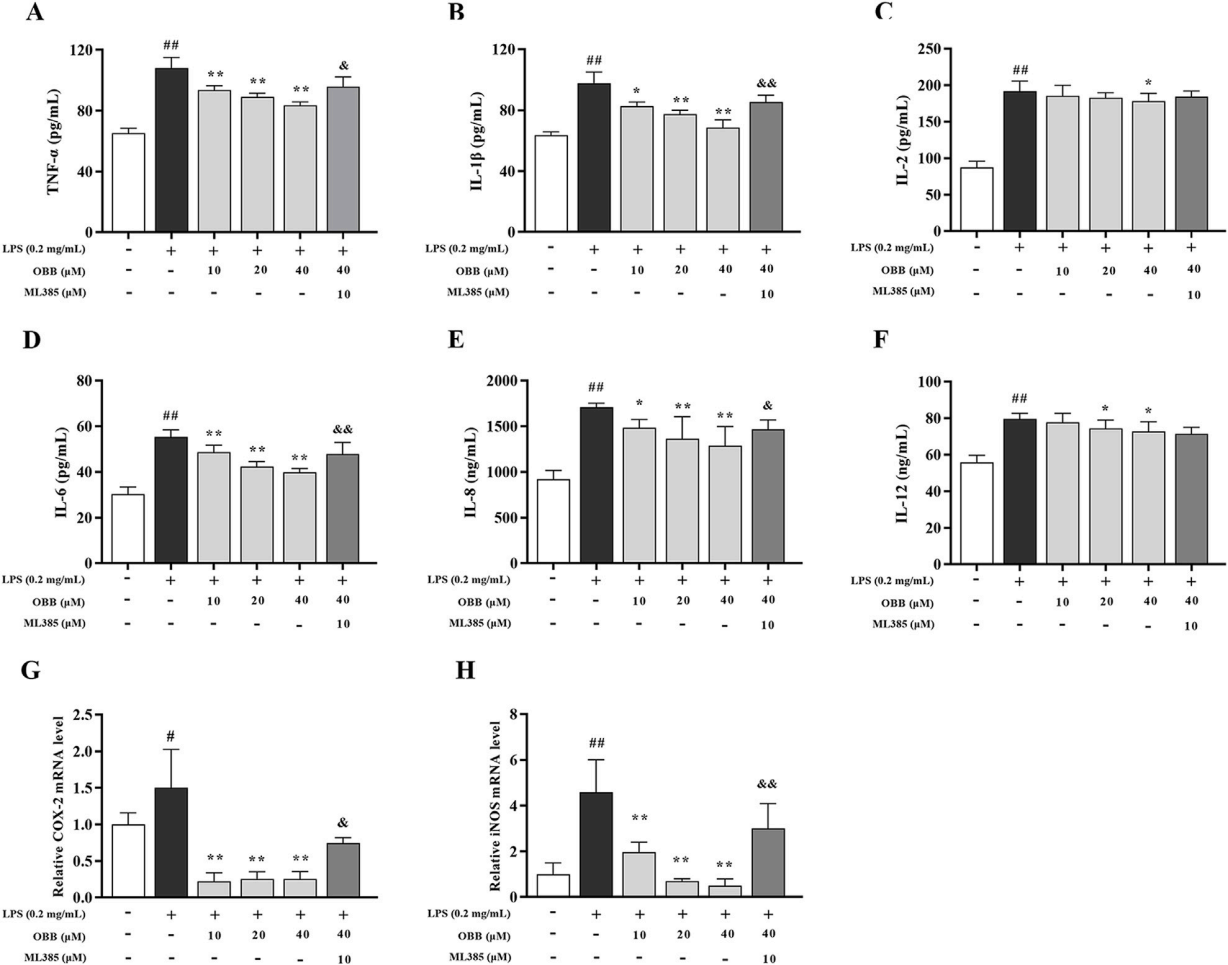
Influence of OBB toward LPS-elicited cellular inflammation. **(A–F)** The protein contents of TNF-α, IL-1β, IL-2, IL-6, IL-8, and IL-12 were detected via ELISA assay. **(G, H)** Relative mRNA expressions of iNOS and COX-2. The data are represented by mean ± SD (n = 3–6). ^#^ *P* < 0.05, ^##^ *P* < 0.01 vs. Control; *P < 0.05, **P < 0.01 vs. LPS group; ^&^ *P* < 0.05, ^&&^ *P* < 0.01 vs. OBB (40 μM) group.

As displayed in Figure 4, compared to the untreated group, LPS disposal enhanced the levels of proinflammatory cytokines comprising TNF-α, IL-1β, IL-2, IL-6, IL-8, and IL-12, and upregulated the mRNA levels of iNOS and COX-2 (*P* < 0.05). OBB treatment could mitigate the inflammatory response compared to the LPS-evoked Caco-2 cells (*P* < 0.05). Furthermore, the improvement of OBB was subsequently reversed by ML385, which indicated that OBB could restrain the inflammatory reaction in LPS-evoked Caco-2 cells by activating Nrf2 signaling.

### 3.5 Effects of OBB on LPS-elicited ROS levels in Caco-2 cells

**FIGURE 5:**
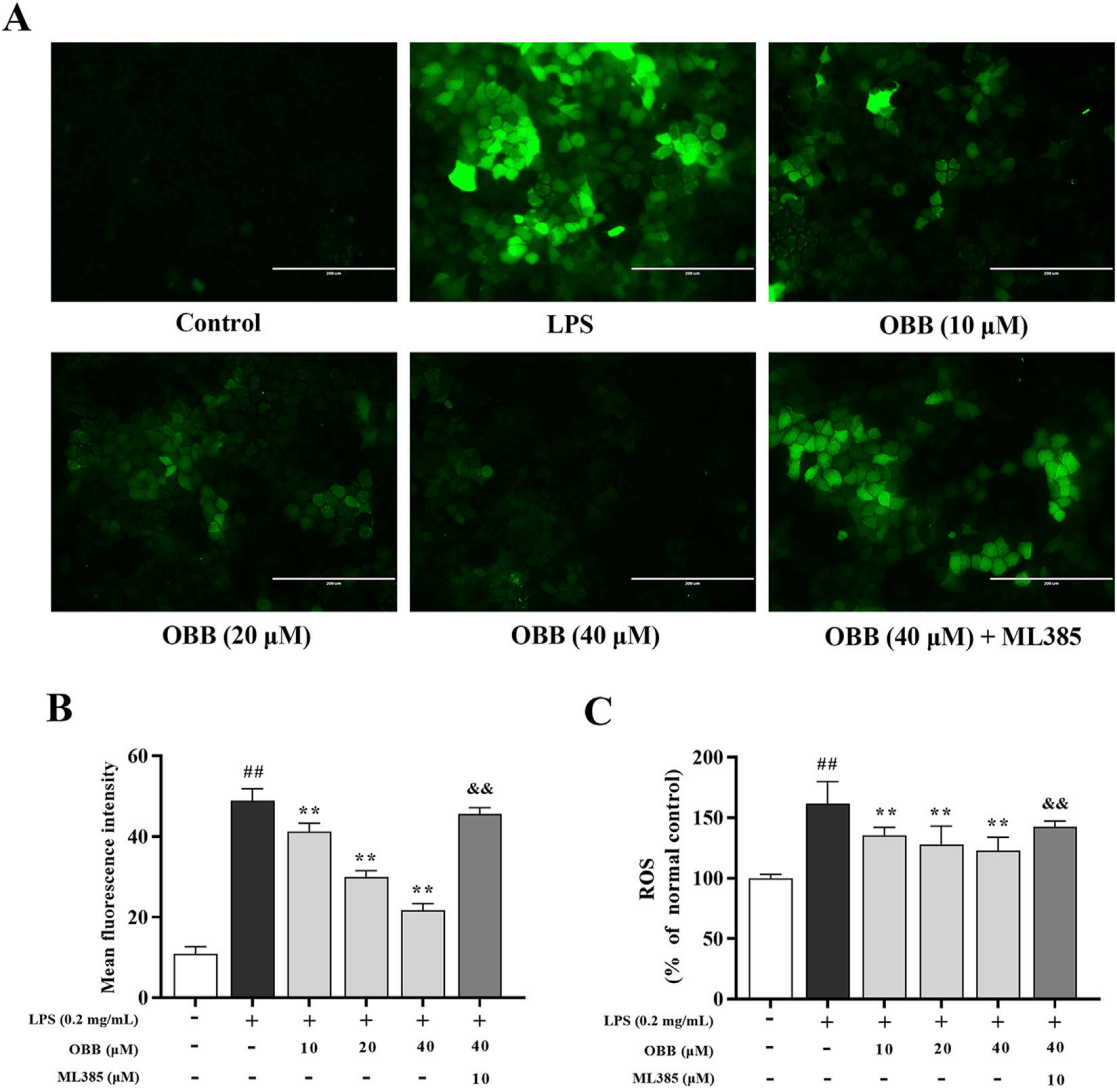
Inspection of cellular ROS via the DCFH-DA test. Influence of OBB on LPS-evoked ROS content was determined using a fluorescent microscope image **(A)**, the average mean fluorescence intensity of ROS **(B)**, and spectrophotometric fluorescence intensity **(C)**. Scale bar represents 200 μm. The data are represented by mean ± SD (n = 3–6). ^##^ *P* < 0.01 vs. Control; **P < 0.01 vs. LPS group; ^&&^ *P* < 0.01 vs. OBB (40 μM) group.

Fluorescence microscopy showed that the green fluorescence intensity for LPS-treated cells in Caco-2 cells was increased compared to the control group (Figures 5A, B). The spectrofluorometry study revealed that the intensity of ROS was increased in the LPS-treated Caco-2 cell (Figure 5C). By contrast, the pretreatment with OBB (10 μM, 20 μM, and 40 μM) inhibited the ROS intensity level in LPS-treated cells, which was reversed by ML385 treatment.

### 3.6 Effects of OBB on LPS-induced apoptosis

**FIGURE 6:**
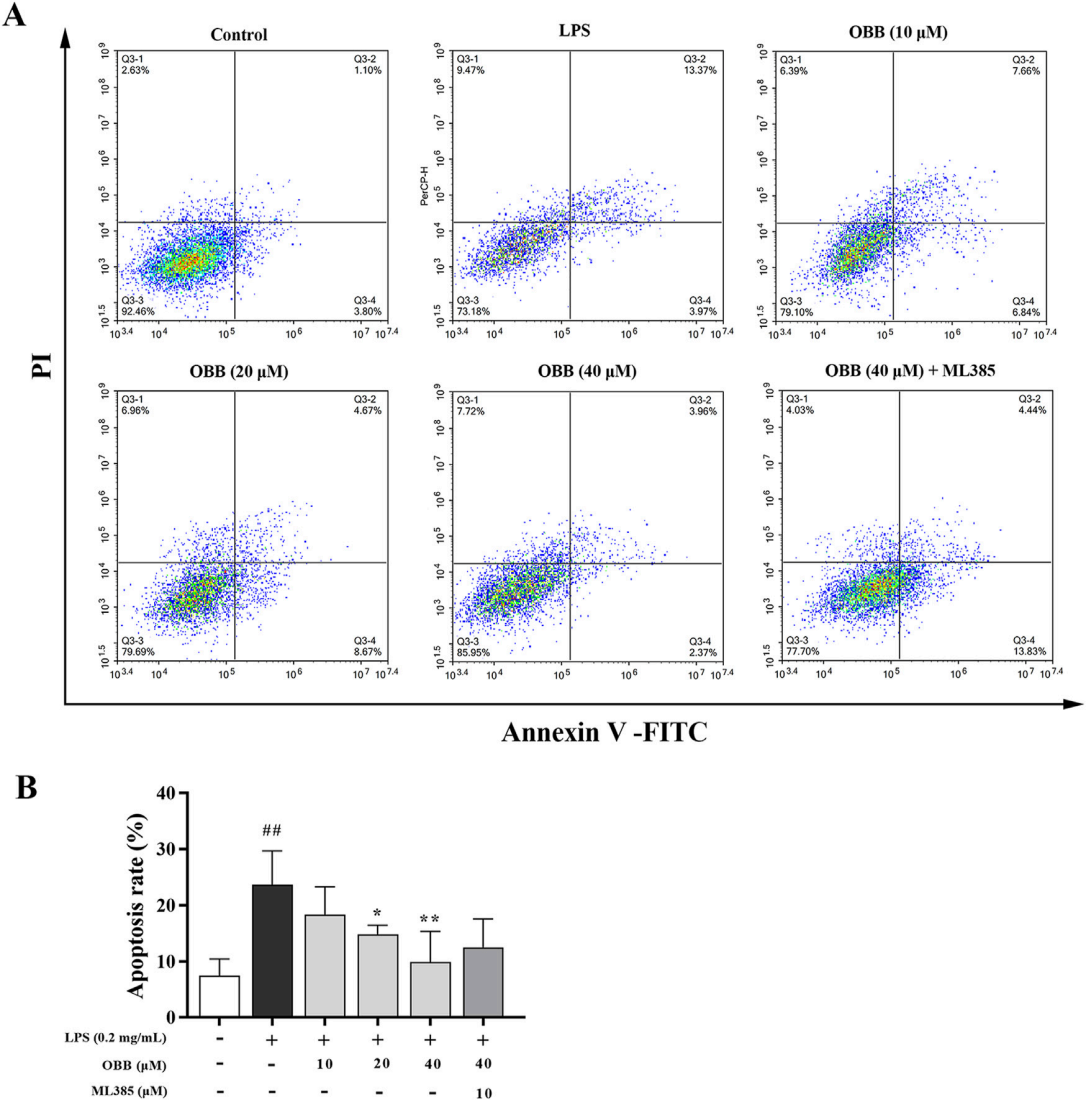
OBB restrains the cellular apoptosis of Caco-2 cells. **(A)** Following OBB intervention for 24 h, cellular apoptosis was inspected using the Annexin V FITC/PI method and flow cytometry. **(B)** Percentage of apoptosis cells. The apoptotic rate was determined by summing the percentages of early apoptosis and late apoptosis. Values are presented by mean ± SD (n = 3). ^##^ *P* < 0.01 vs. Control; *P < 0.05, **P < 0.01 vs. LPS group.

As displayed in Figure 6, LPS significantly elevated cellular apoptosis compared to the normal group (*P* < 0.01). The OBB remedy (10 μM, 20 μM, and 40 μM) reduced the apoptosis rate dose-dependently compared to LPS alone (*P* < 0.05). The anti-apoptosis role of OBB was reversed by ML385 treatment.

### 3.7 Influences of OBB on Nrf2/NF-κB signaling pathways in LPS-elicited Caco-2 cellular damage

**FIGURE 7:**
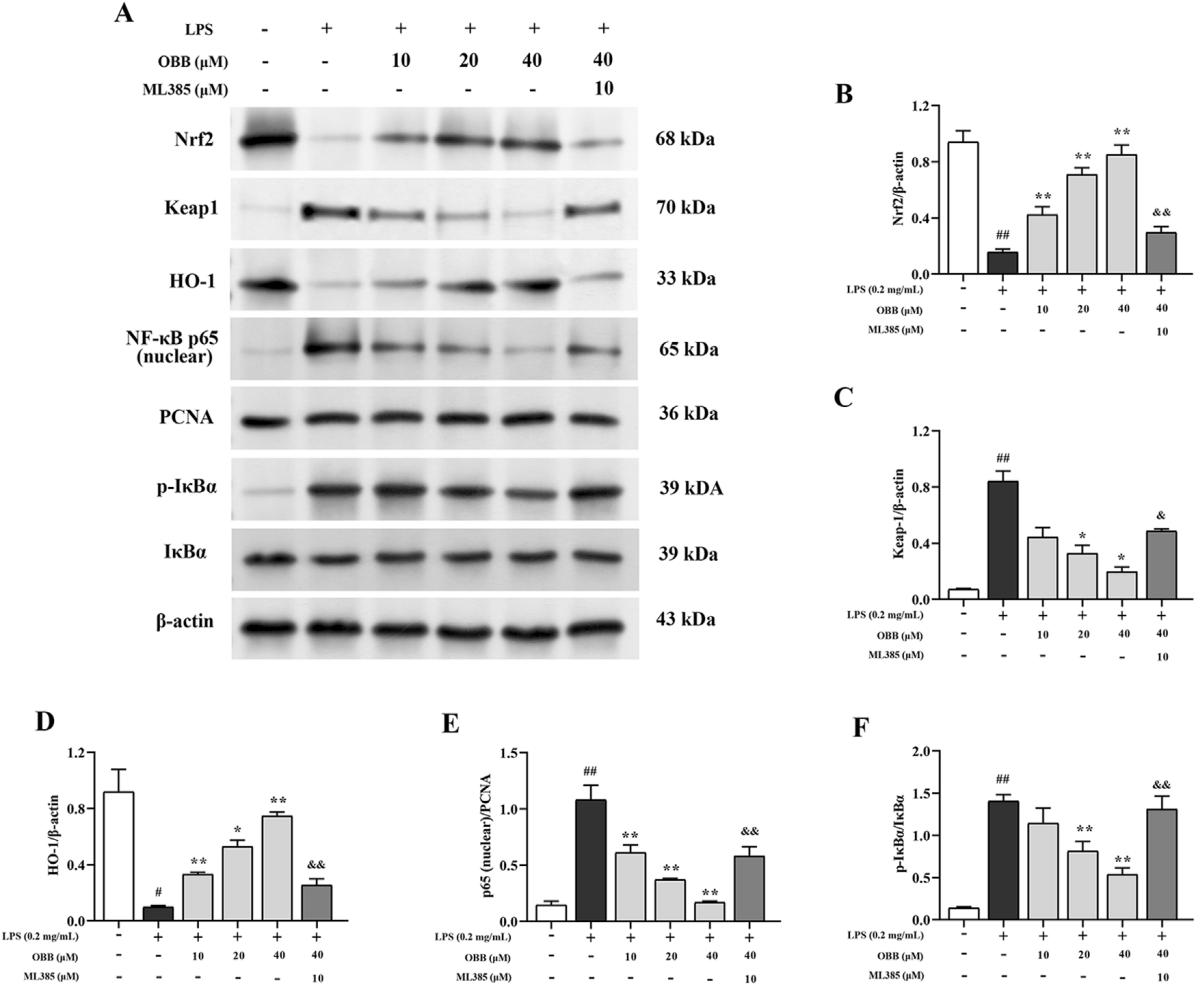
Impacts of OBB on Nrf2/NF-κB signal paths in LPS-elicited cellular damage in Caco-2 cells. The protein expressions of Nrf2, Keap1, HO-1, NF-κB p65 (nuclear), IκBα, and p-IκBα in Caco-2 cells were detected via Western blot. The data are represented by mean ± SD (n = 3). ^##^ *P* < 0.01 vs. Control; *P < 0.05, **P < 0.01 vs. LPS group; ^&^ *P* < 0.05, ^&&^ *P* < 0.01 vs. OBB (40 μM) group.

The expressions of Nrf2, Keap1, HO-1, NF-κB p65 (nuclear), IκBα, and p-IκBα were inspected via Western blotting. As presented in Figure 7, the expressions of Keap1, nuclear p65, and p-IκBα were enhanced in LPS-elicited cells compared to the control group (*P* < 0.05). OBB (10 μM, 20 μM, and 40 μM) treatment lowered the levels of Keap1, nuclear p65, and p-IκBα/IκBα and elevated the expressions of Nrf2 and HO-1 in a dose-dependent manner (*P* < 0.05). However, the regulation of OBB on the above proteins was reversed by the Nrf2 inhibitor, ML385. The proofs indicated that OBB regulated the Nrf2/NF-κB signal paths to prevent LPS-elicited cellular damage in Caco-2 cells.

## 4 Discussion

Colitis is a chronic inflammatory gastrointestinal disease characterized by periods of remission and relapse. It affects the quality of life for many people in advanced nations worldwide (Cichewicz et al., 2023). The etiology of colitis is not yet fully comprehended, and present therapy is very unsatisfactory. It is highly ineffectual and also may cause severe adverse reactions among some patients (Panés et al., 2024; Zhang et al., 2024). Thus, novel interventions and therapeutical methods are required to fight inflammatory diseases.

Our preceding research has proved that OBB could ameliorate inflammatory reactions in DSS or TNBS-induced murine colitis. However, the role of OBB toward inflammatory reactions in IECs in damaged states is still unclear. The evidence of CCK-8 indicated that OBB reduced cellular activity at a 50 μM concentration compared to untreated cells (*P* < 0.05) after 24 h of culture. It indicated that OBB possessed no toxicity to Caco-2 cells after 24 h at concentrations less than 100 μM.

The required role of the enteric epithelial barrier is the intake of electrolytes and nutrients, but it also serves as a semipermeable barrier that exerts a crucial action in defending the body against microbiological pathogens and possibly toxic materials (Bergheim and Moreno-Navarrete, 2024; Yao et al., 2024). Dysfunction of the enteric epithelial barrier increases gut penetrability. It causes many kinds of serious enteric inflammatory diseases, including inflammatory bowel disease (IBD), necrotizing enterocolitis, irritable bowel syndrome (IBS), and metabolic syndrome (Barbara et al., 2021; Li H. L. et al., 2024). The appropriate function of the enteric epithelial barrier is kept by epithelial cells and intercellular TJs. TJs consist of a set of transmembranous proteins (occludins and claudins), peripheric membranous proteins (zonula occludens), and adjacent adherens junctions (E-cadherin), which interact with each other and constitute a selecting barrier through interplaying with adjacent cells (Peng et al., 2023). Former studies have proved that damaging TJs results in the increased permeability of the enteric epithelial barrier (Horowitz et al., 2023). Thus, TJ proteins are vital for maintaining the integrity of the enteric epithelial barrier.

We found that OBB ameliorated the enteric epithelial barrier dysfunction aroused by proinflammatory factors *in vitro*. Caco-2 single-cell layers treated with or without OBB for 2 h were subjected to 0.2 mg/mL LPS for a further 24 h. The results indicated that LPS injured epithelial barrier function and induced an abnormal inflammatory response, which is in agreement with the preceding investigation (Nathani et al., 2023; Zheng et al., 2022). The administration of OBB ameliorated the decline in TEER and the rise in intercellular penetrability in a dose-dependent manner compared to sole LPS intervention. These results suggested that OBB defends the barrier function of Caco-2 cells from LPS-evoked injury. In addition, the Western blot test showed that the protein expression of ZO-1, E-cadherin, and occludin was down-modulated in Caco-2 cells subjected to 0.2 mg/mL LPS. Administration of OBB up-modulated expression of the TJ proteins compared to sole LPS intervention. The immunofluorescent result of TJ proteins showed that OBB mitigated LPS-elicited changes in the distribution of TJ proteins. The results indicated that OBB exerts a main action in maintaining epithelial barrier function by mitigating LPS-elicited variations in the expression and distribution of TJ proteins.

Enteric inflammation was revealed to be related to enteric epithelial barrier damage. Many studies have proved that enteric epithelial inflammation could injure TJs by down-modulating the level of TJ proteins via inflammatory signal transduction (Zheng et al., 2023). NF-κB is a crucial transcriptional factor that adjusts inflammatory reactions by regulating the transcription of diversified cell genes and has been authenticated as a therapeutical target for the remedy of enteric inflammation and IBD (Amirshahrokhi and Imani, 2023; Tsopmejio et al., 2022). LPS exposure evokes IκBα phosphorylation, bringing about IκBα degradation, which leads to the release and translocation of NF-κB from the cytoplasm to the nucleus. Inside the nucleus, p65 upmodulates the transcription of proinflammatory mediators and cytokines such as COX-2, TNF-α, iNOS, and IL-6 (Ghasemi-Dehnoo et al., 2023; Kim et al., 2023). Studies have indicated that OBB suppressed the inflammatory response by restraining the NF-κB signal path. This study identified the NF-κB signaling pathway as a crucial mechanism underlying the regulation of the intestinal monolayer barrier by OBB. The results showed that LPS-evoked NF-κB excitation in Caco-2 cells was restrained by OBB. Furthermore, OBB reverted the up-modulation of LPS-elicited IL-6, iNOS, TNF-α, and COX-2 expression in a dose-dependent mode at the mRNA and protein levels.

Enteric epithelial oxidant stress, characterized by superfluous ROS generation, has been found to break the enteric epithelial barrier and induce mucosa disorders (Zeng et al., 2023). Additionally, elevated production of ROS can accelerate inflammatory reactions in the intestinal tract, leading to the pathogeny of IBD (Zhang et al., 2023). Nrf2 is regarded as the master cellular sensor of oxidant stress. Nrf2 is a basic-leucine zipper transcription factor that exists in the cytoplasm, binding to Keap1 in homeostatic circumstances (Ulasov et al., 2022). When cells are subjected to oxidant stress, Nrf2 dissociates from Keap1 and translocates into the nucleus, where it binds to the antioxidant-responsive element (ARE), bringing about the increased expression of antioxidative genes involving HO-1 and NQO-1 (Li J. C. et al., 2021). Earlier studies showed that the Nrf2/HO-1 path has assumed one of the upriver molecules considered targets for anti-NF-κB-evoked inflammation (Yan et al., 2021). Targeted activation of Nrf2 signaling can effectively regulate the intestinal mucosal barrier function (Dong et al., 2023). In the present research, OBB intervention restrained LPS-evoked down-modulation of Nrf2 and HO-1 and up-modulation of NF-κB. It is critical that the Nrf2 depressor ML385 abolished the protection of OBB on Caco-2 cells, partly counteracted OBB-elicited excitation of Nrf2 signaling, and suppressed the NF-κB signal path, indicating that OBB restrained the NF-κB-reliant inflammatory reaction via motivating the Nrf2/HO-1 path. Thus, the protection of OBB against LPS-elicited enteric epithelial barrier damage was partly modulated by excitation of the Nrf2 signal and suppression of the NF-κB signal path. By confirming the excitation of the antioxidant adaptive response and the restraint of inflammation, the Nrf2 and NF-κB signal paths increase the protection caused by OBB against LPS-evoked inflammation in the enteric epithelia.

## 5 Conclusion

**FIGURE 8:**
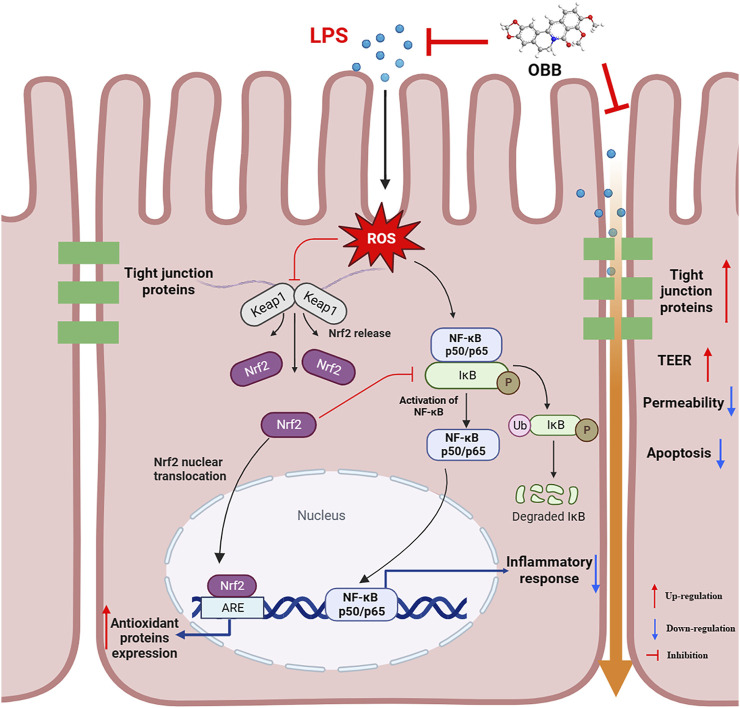
Proposed mechanism of OBB amelioration of LPS-induced barrier function dysfunction, oxidative stress, and inflammatory reaction in Caco-2 cells.

In conclusion, the present study indicated that OBB plays a protective effect on LPS-evoked colitis by repressing oxidative damage, inflammatory reactions, and apoptosis and increasing intestinal TJ proteins in Caco-2 cells. Furthermore, Nrf2 was important in the development of colitis, and OBB was able to trigger Nrf2 signaling and inhibit the Nrf2-initiated NF-κB path to achieve its regulatory effect in human Caco-2 cells (Figure 8). Collectively, this evidence may help ensure the employment of OBB as a therapeutical method for inflammation-related disease control in patients with colitis.

## Data Availability

The original contributions presented in the study are included in the article/Supplementary Material, further inquiries can be directed to the corresponding author.
