# Supplementary figures and images for: Oxyberberine alleviates lipopolysaccharide-induced intestinal barrier disruption and inflammation in human colonic Caco-2 cells in vitro
Source: Front Pharmacol. 2025 Jan 7;15:1496874. doi: 10.3389/fphar.2024.1496874 (PMC11747431; doi:10.3389/fphar.2024.1496874)

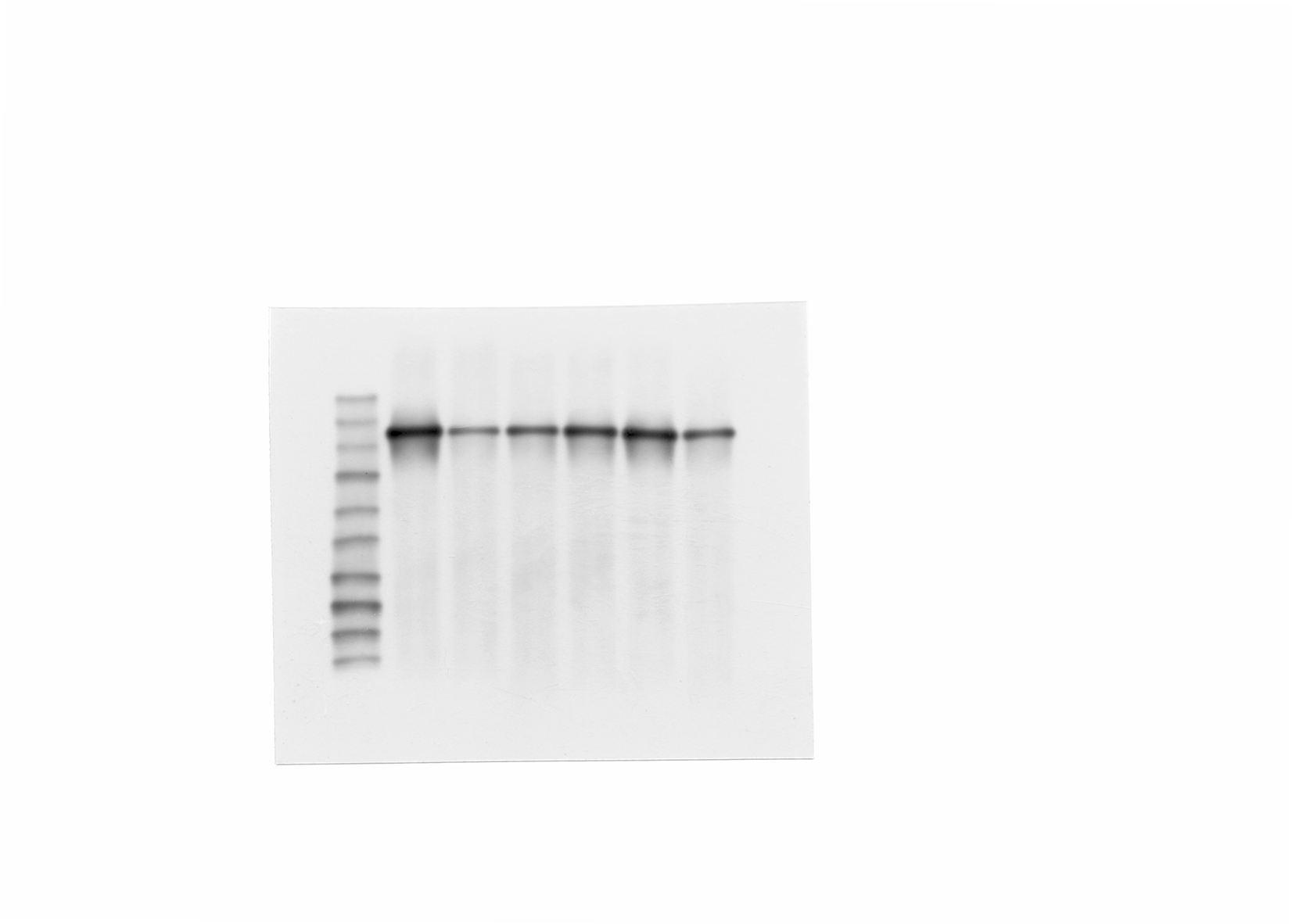

Supplement: Supplementary file 1 [file DataSheet1.zip › Image of the original Western blots/E-cadherin 120 kDa 1.tif]

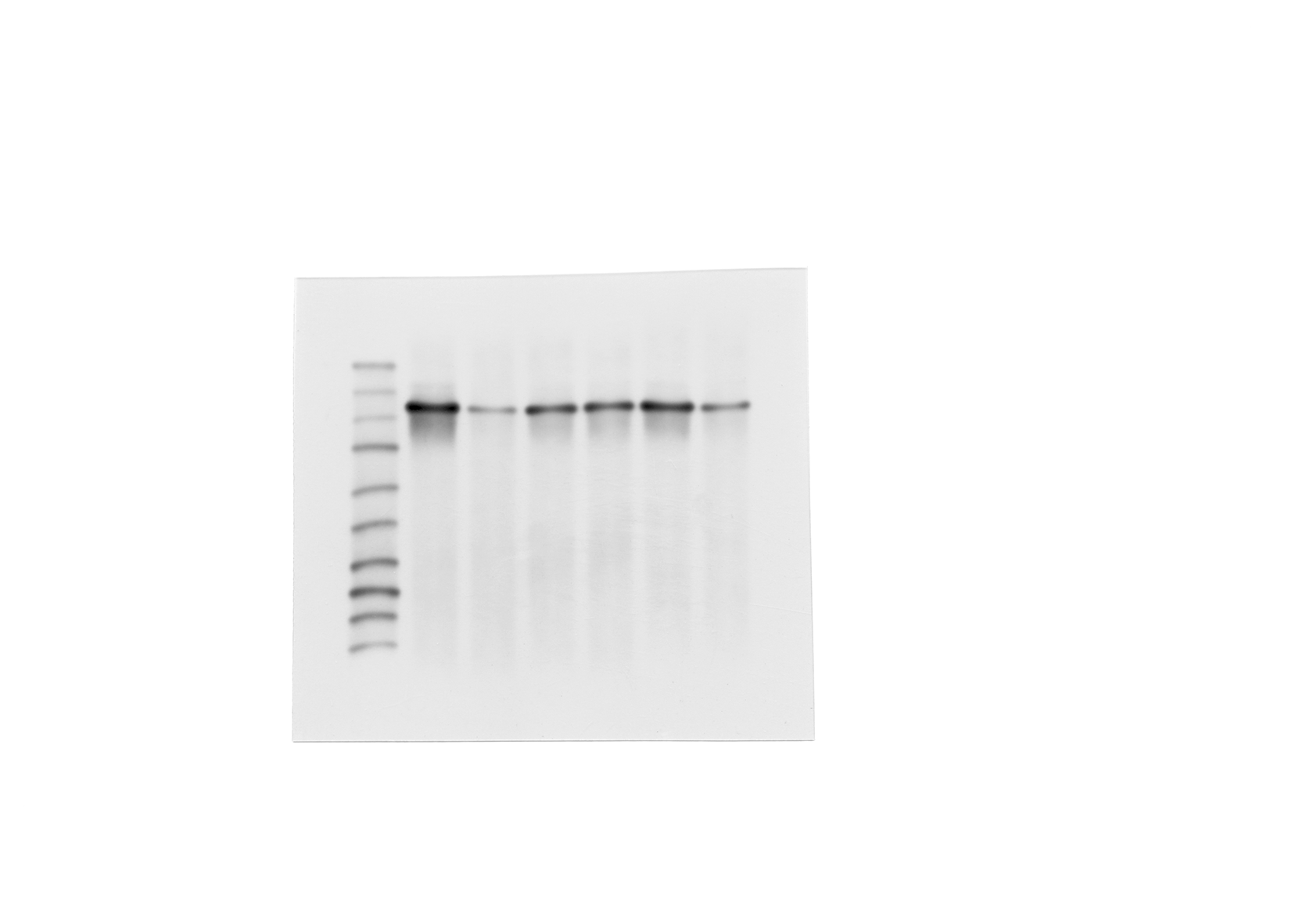

Supplement: Supplementary file 1 [file DataSheet1.zip › Image of the original Western blots/E-cadherin 120 kDa 2.tif]

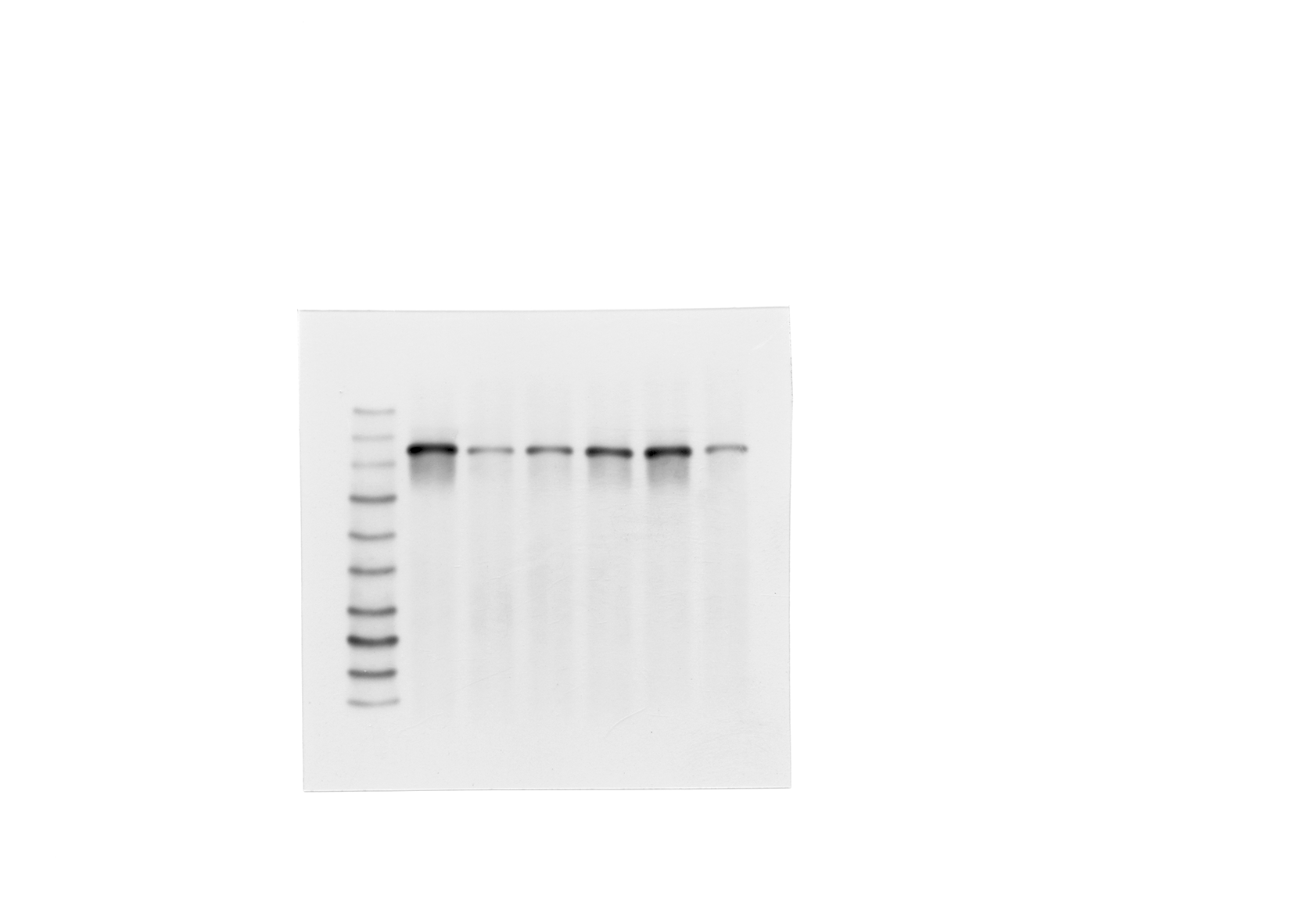

Supplement: Supplementary file 1 [file DataSheet1.zip › Image of the original Western blots/E-cadherin 120 kDa 3.tif]

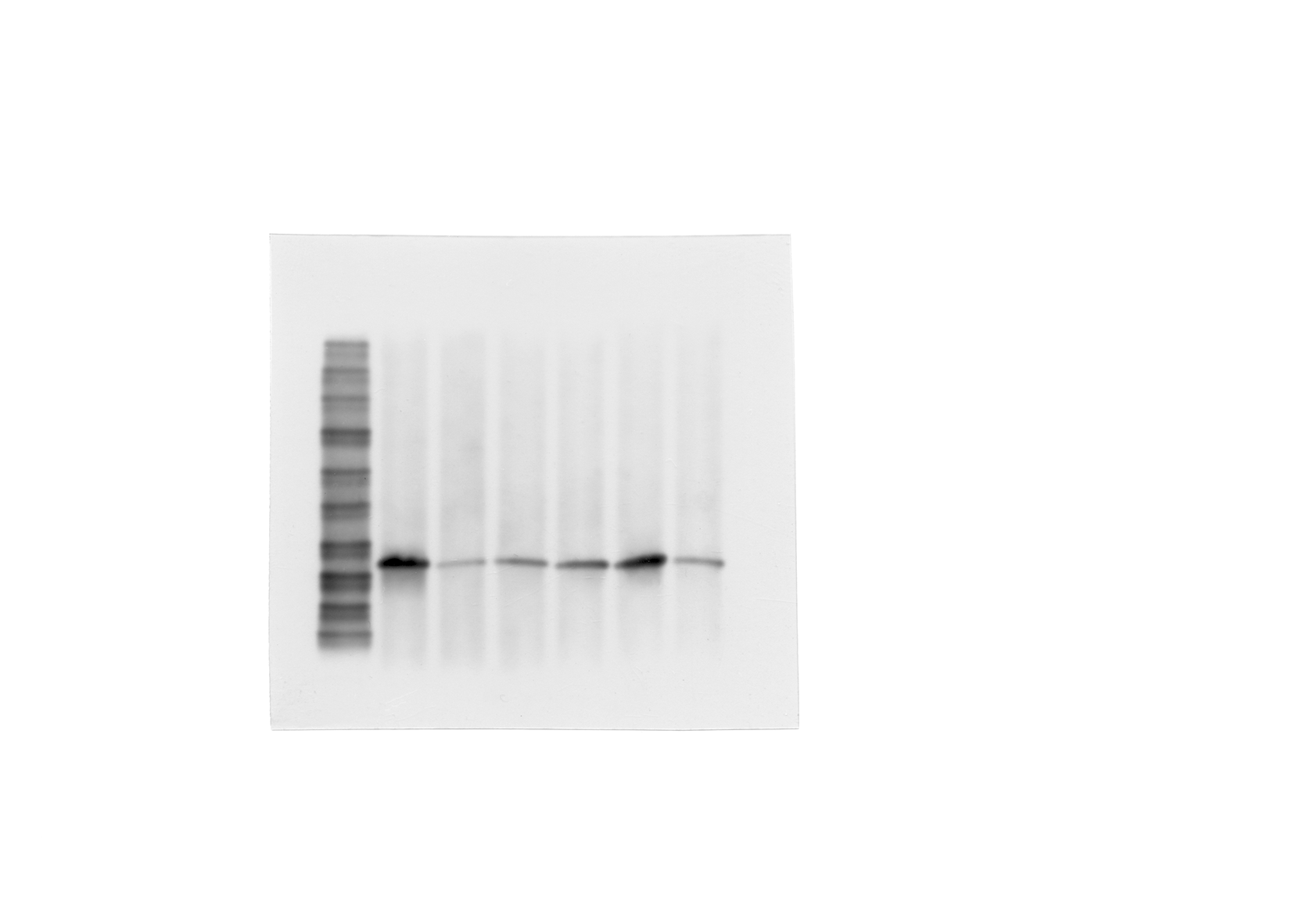

Supplement: Supplementary file 1 [file DataSheet1.zip › Image of the original Western blots/HO-1 33 kDa 1.tif]

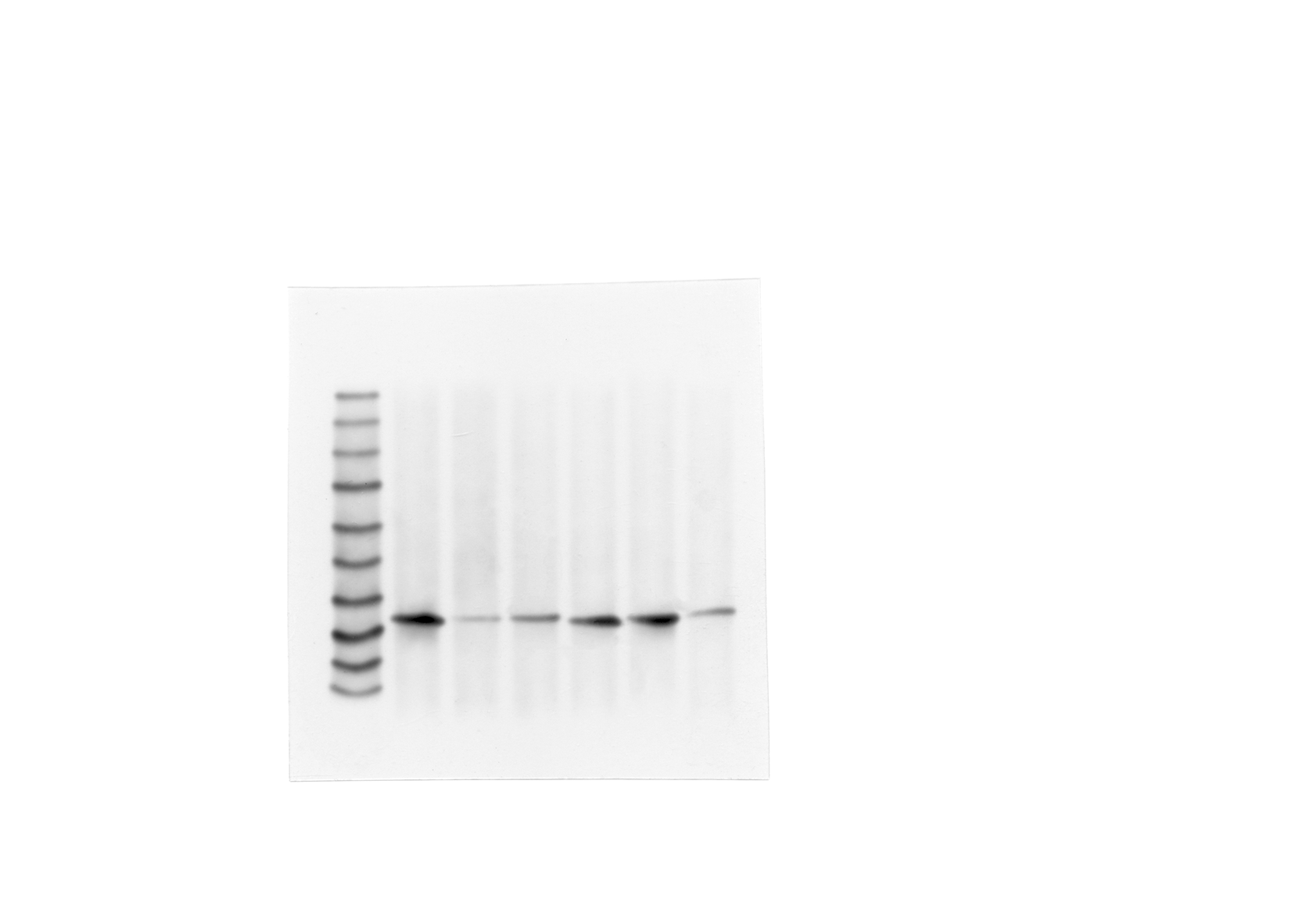

Supplement: Supplementary file 1 [file DataSheet1.zip › Image of the original Western blots/HO-1 33 kDa 2.tif]

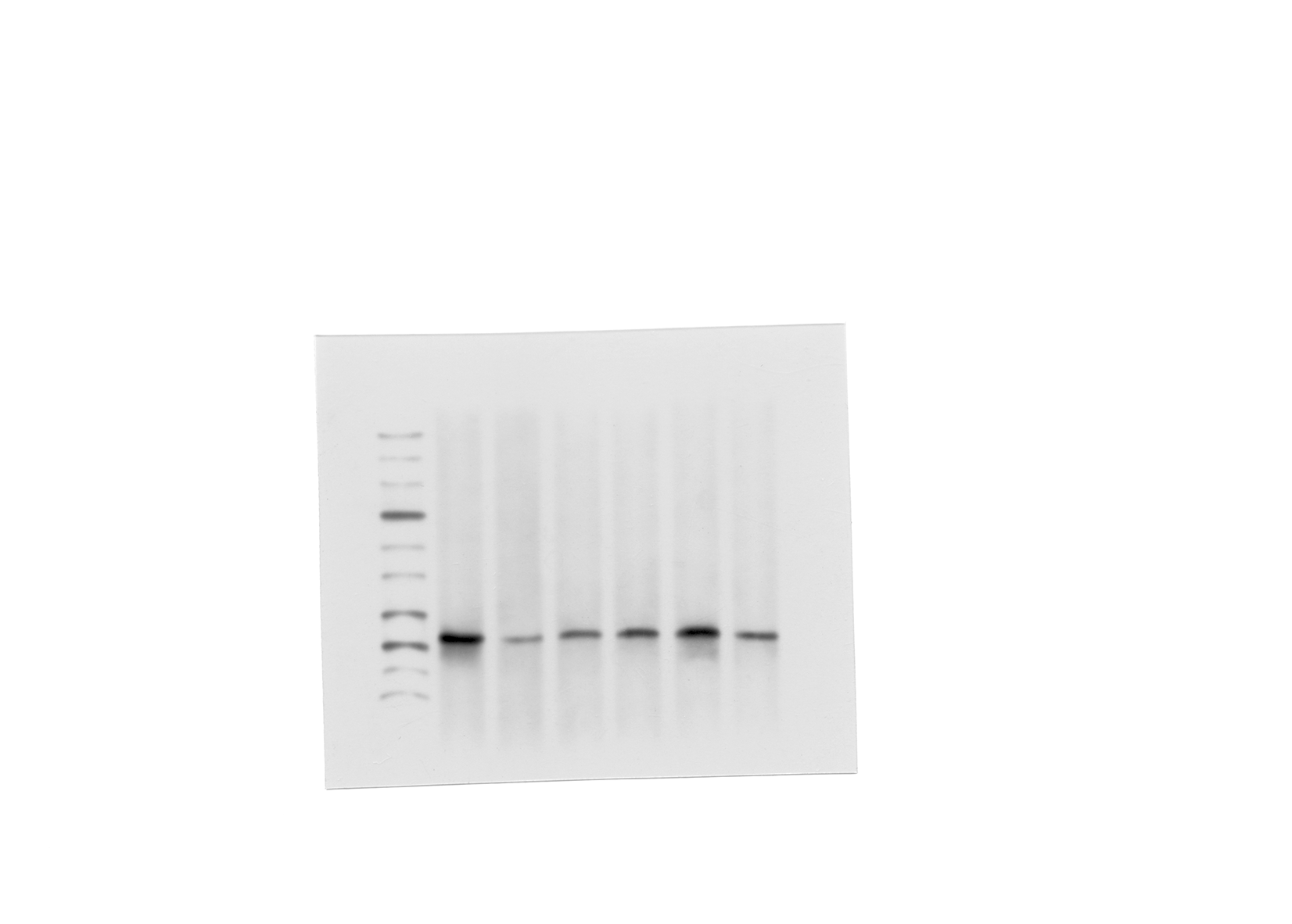

Supplement: Supplementary file 1 [file DataSheet1.zip › Image of the original Western blots/HO-1 33 kDa 3.tif]

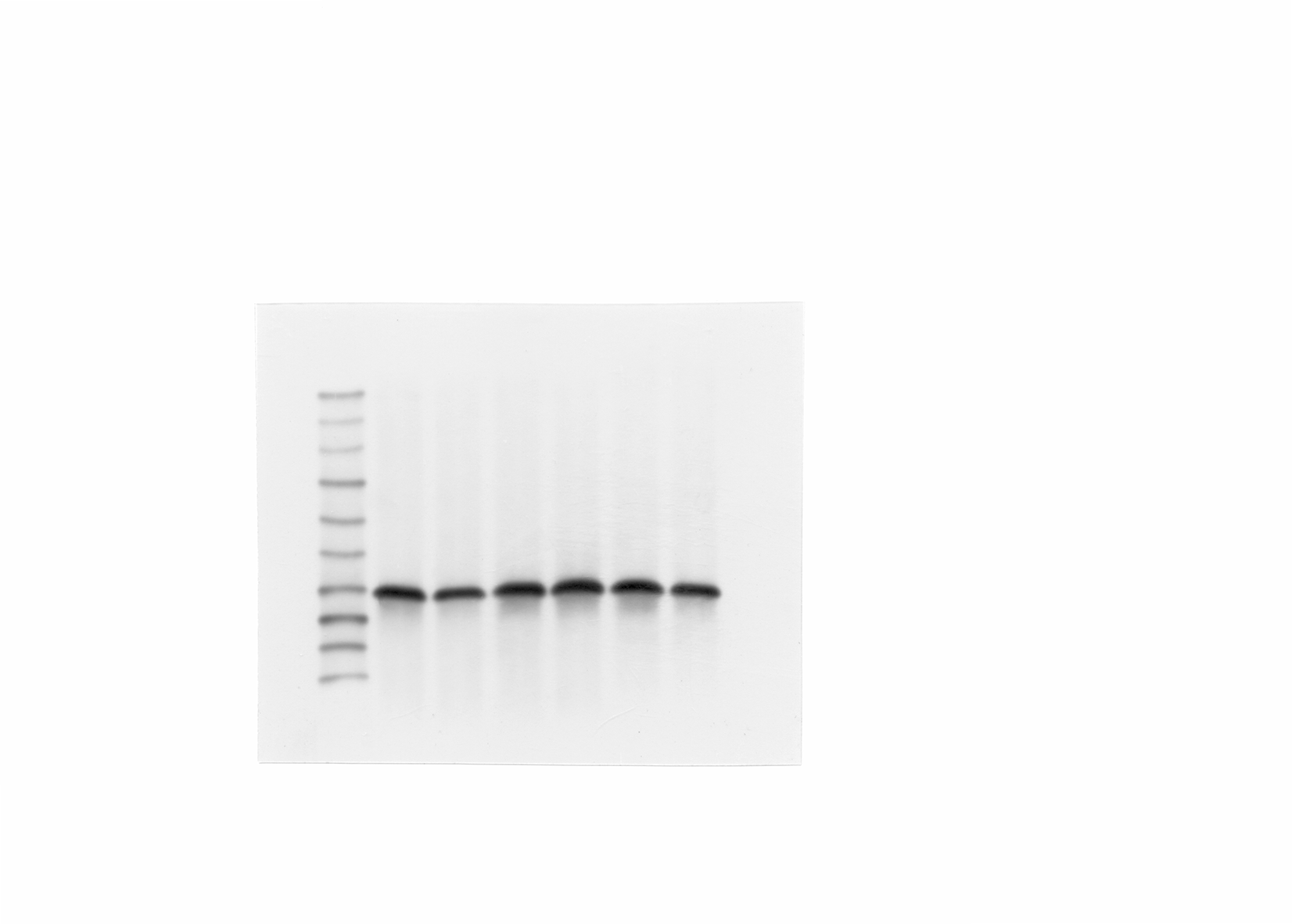

Supplement: Supplementary file 1 [file DataSheet1.zip › Image of the original Western blots/Ia╩Ba┴ 39 kDa 1.tif]

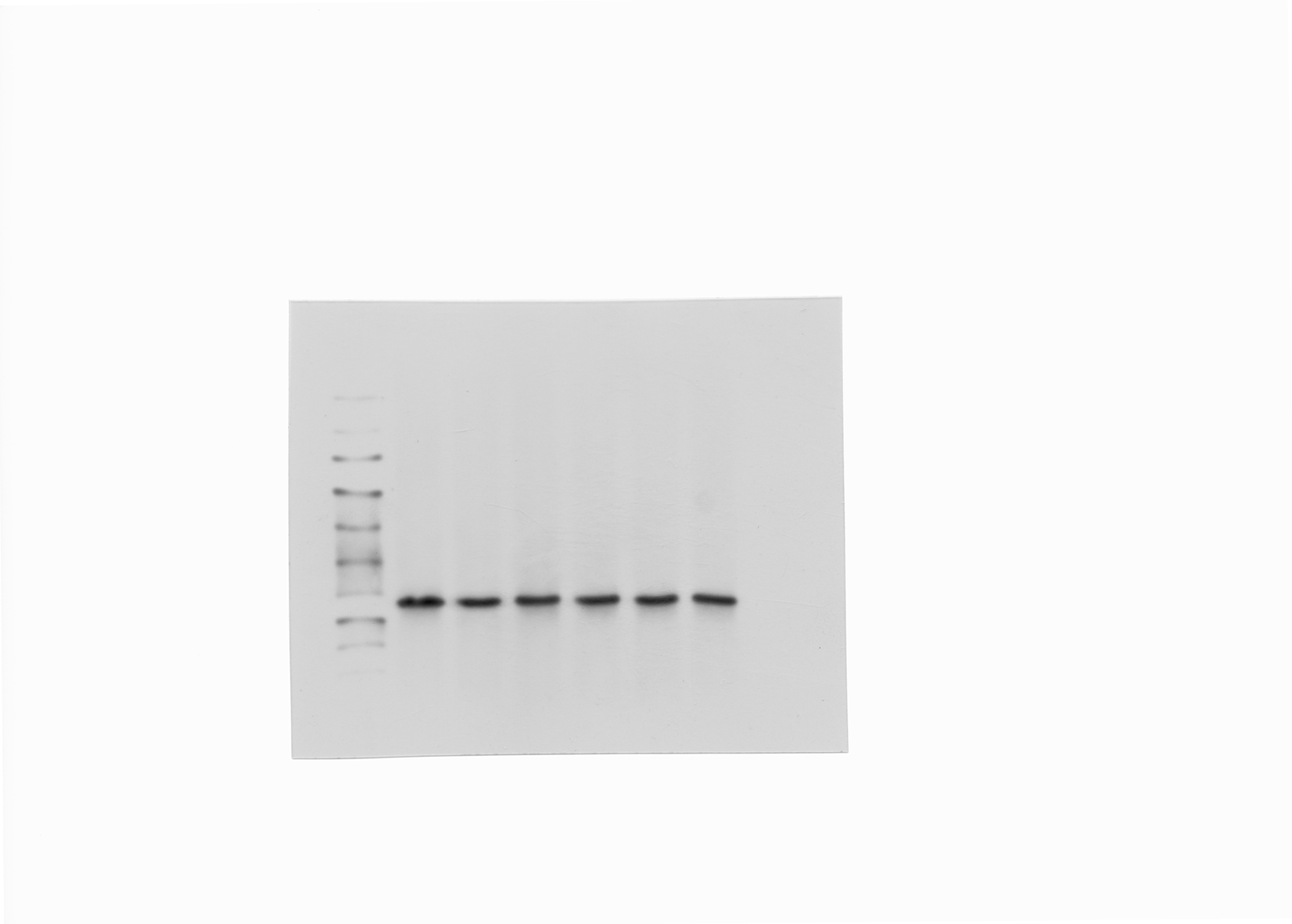

Supplement: Supplementary file 1 [file DataSheet1.zip › Image of the original Western blots/Ia╩Ba┴ 39 kDa 2.tif]

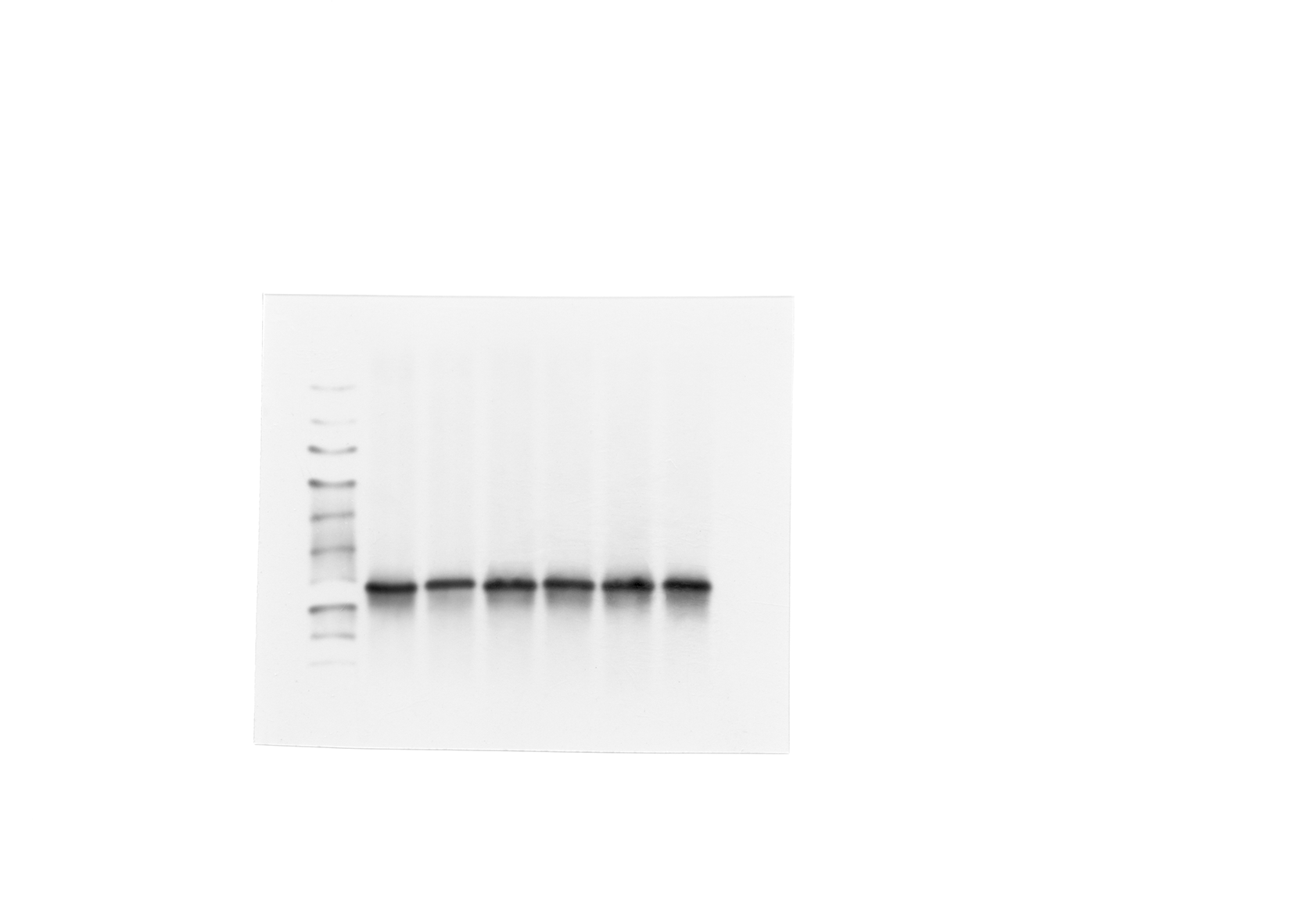

Supplement: Supplementary file 1 [file DataSheet1.zip › Image of the original Western blots/Ia╩Ba┴ 39 kDa 3.tif]

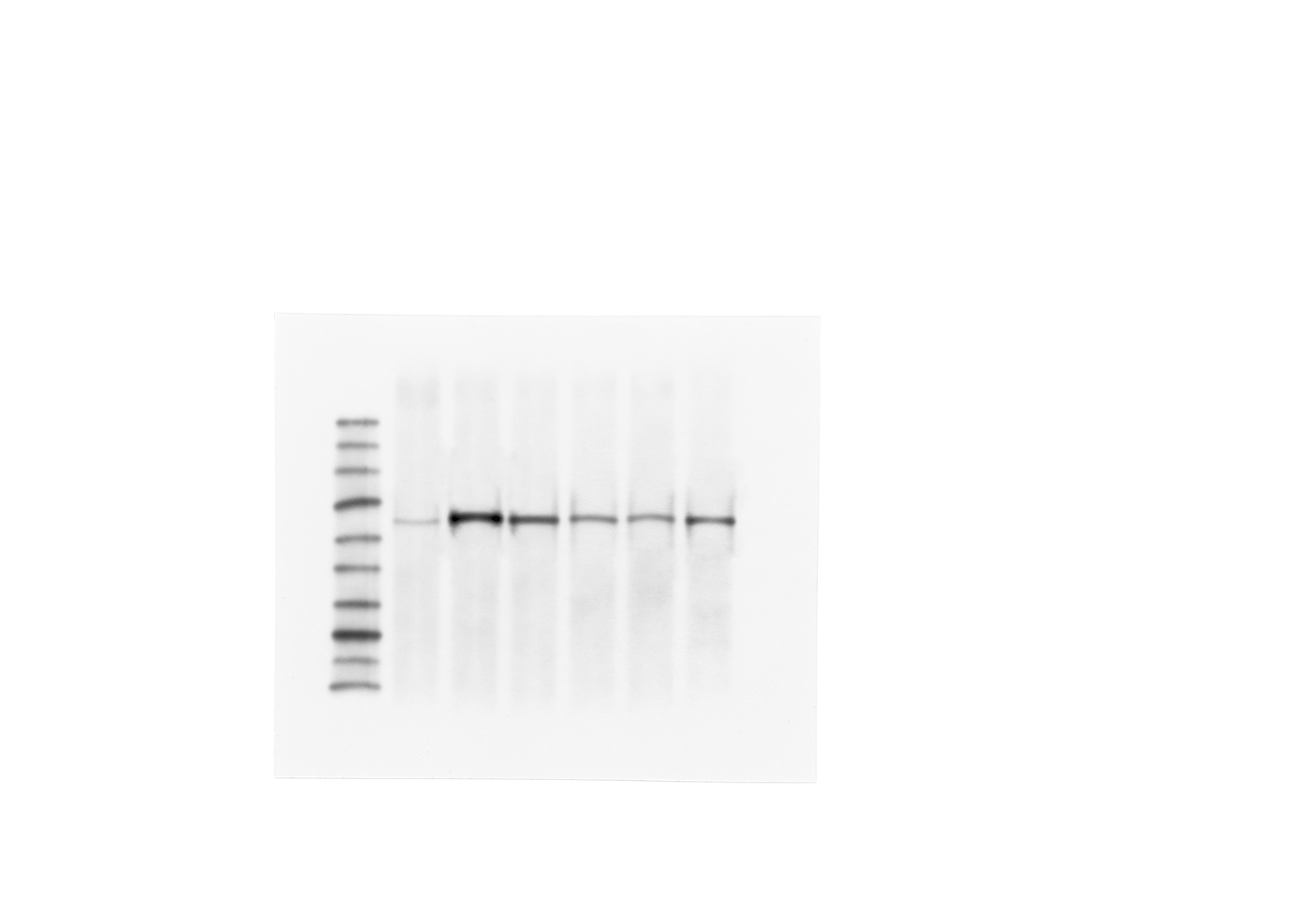

Supplement: Supplementary file 1 [file DataSheet1.zip › Image of the original Western blots/Keap-1 70 kDa 1.tif]

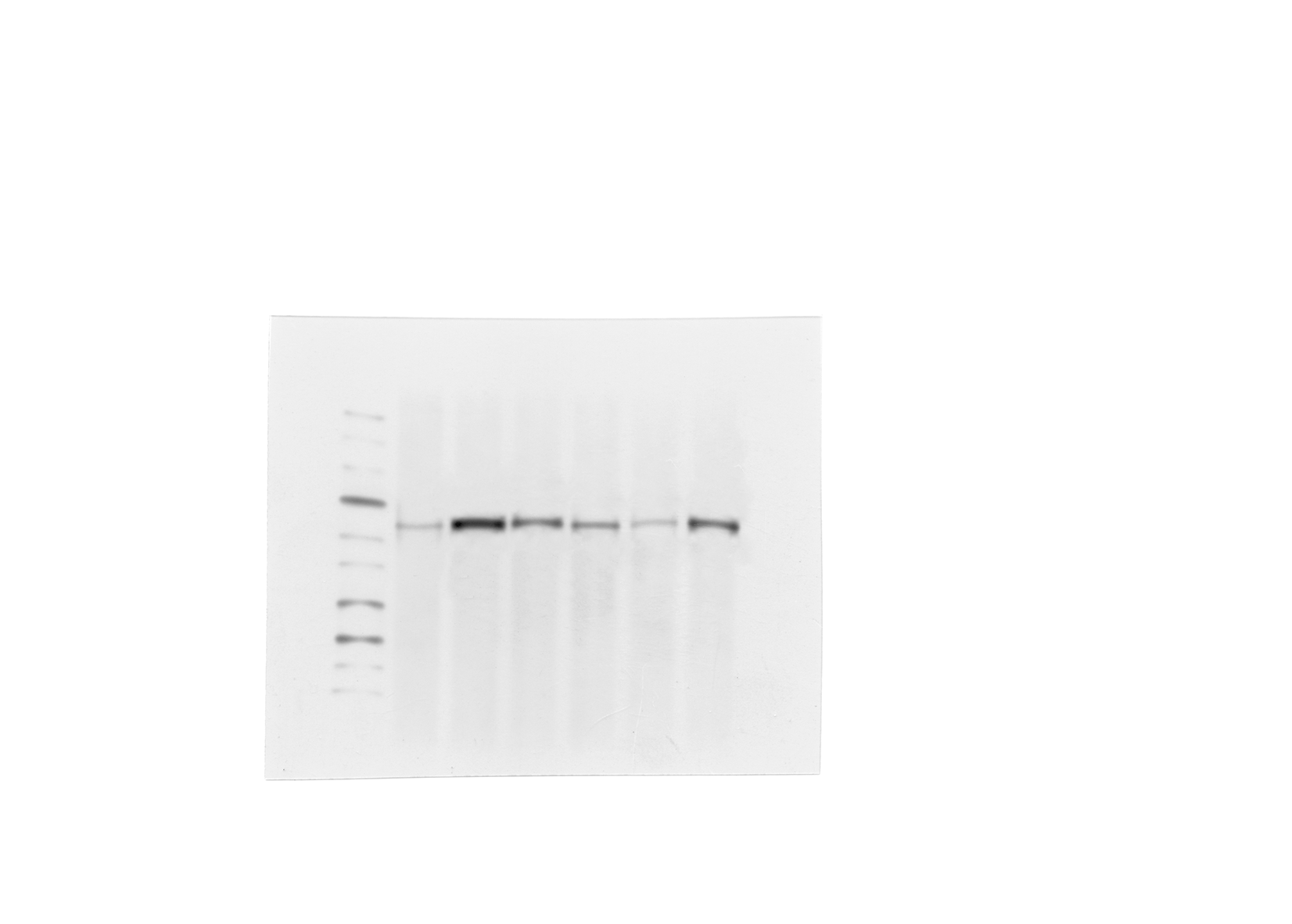

Supplement: Supplementary file 1 [file DataSheet1.zip › Image of the original Western blots/Keap-1 70 kDa 2.tif]

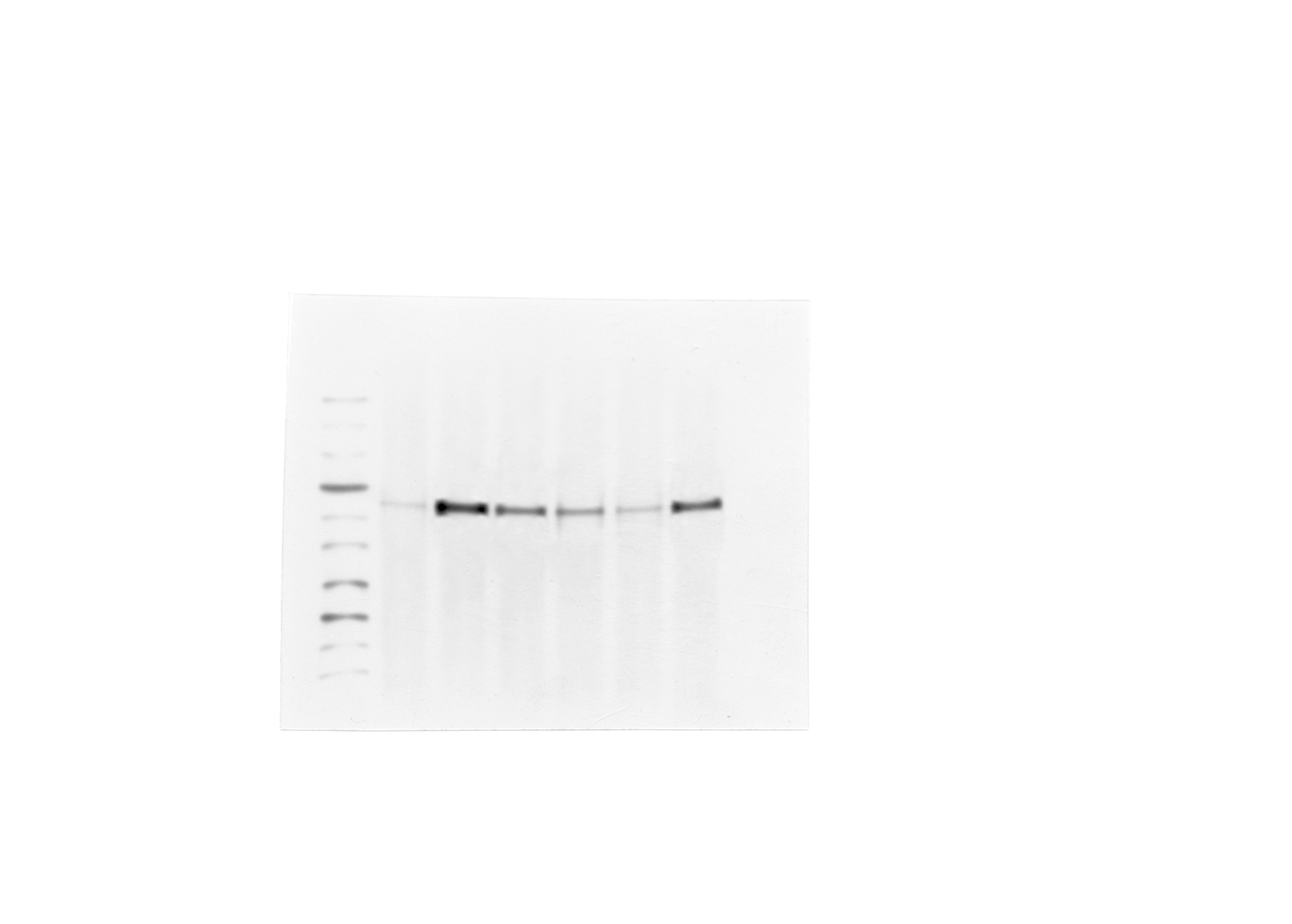

Supplement: Supplementary file 1 [file DataSheet1.zip › Image of the original Western blots/Keap-1 70 kDa 3.tif]

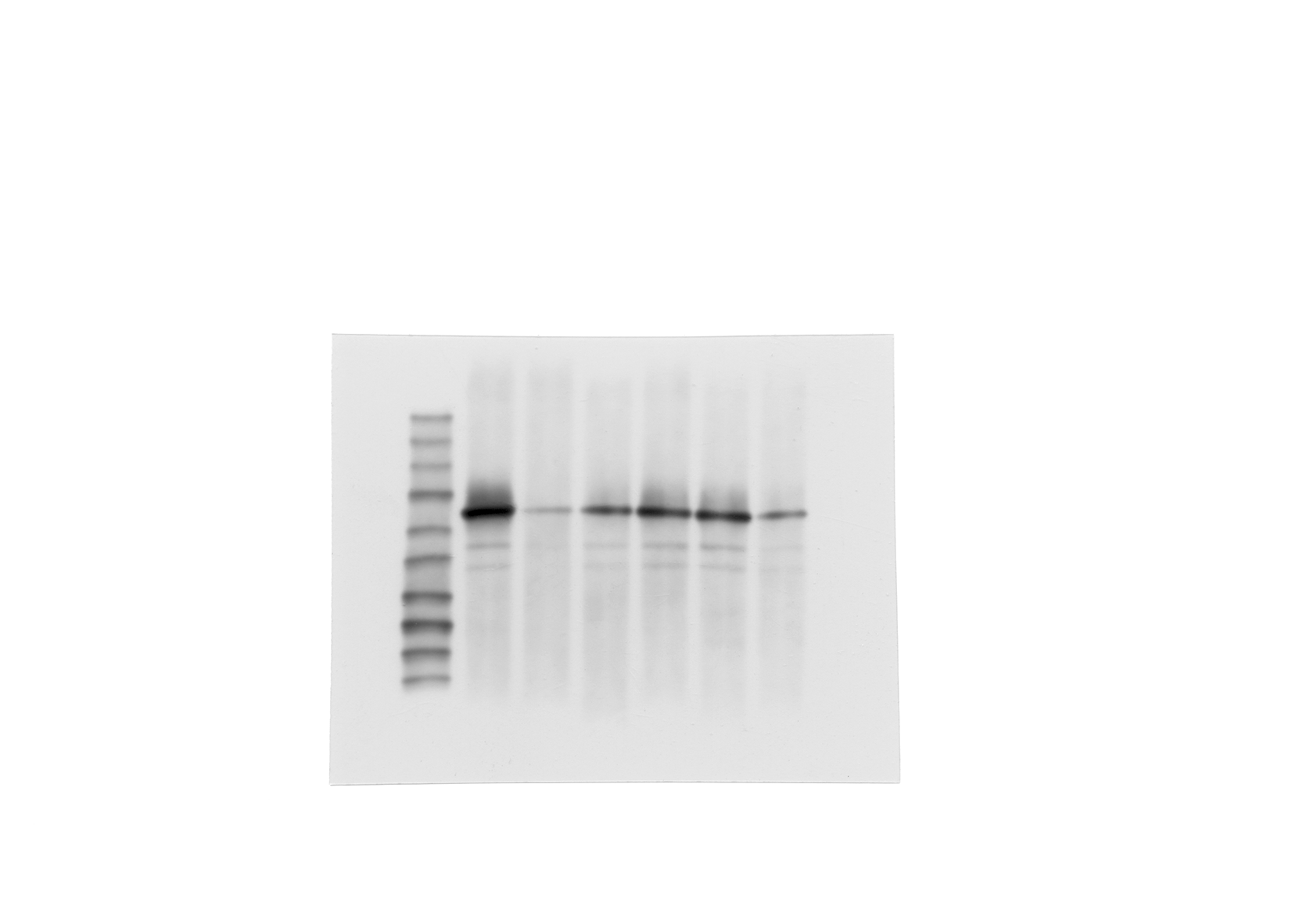

Supplement: Supplementary file 1 [file DataSheet1.zip › Image of the original Western blots/Nrf2 68 kDa 1.tif]

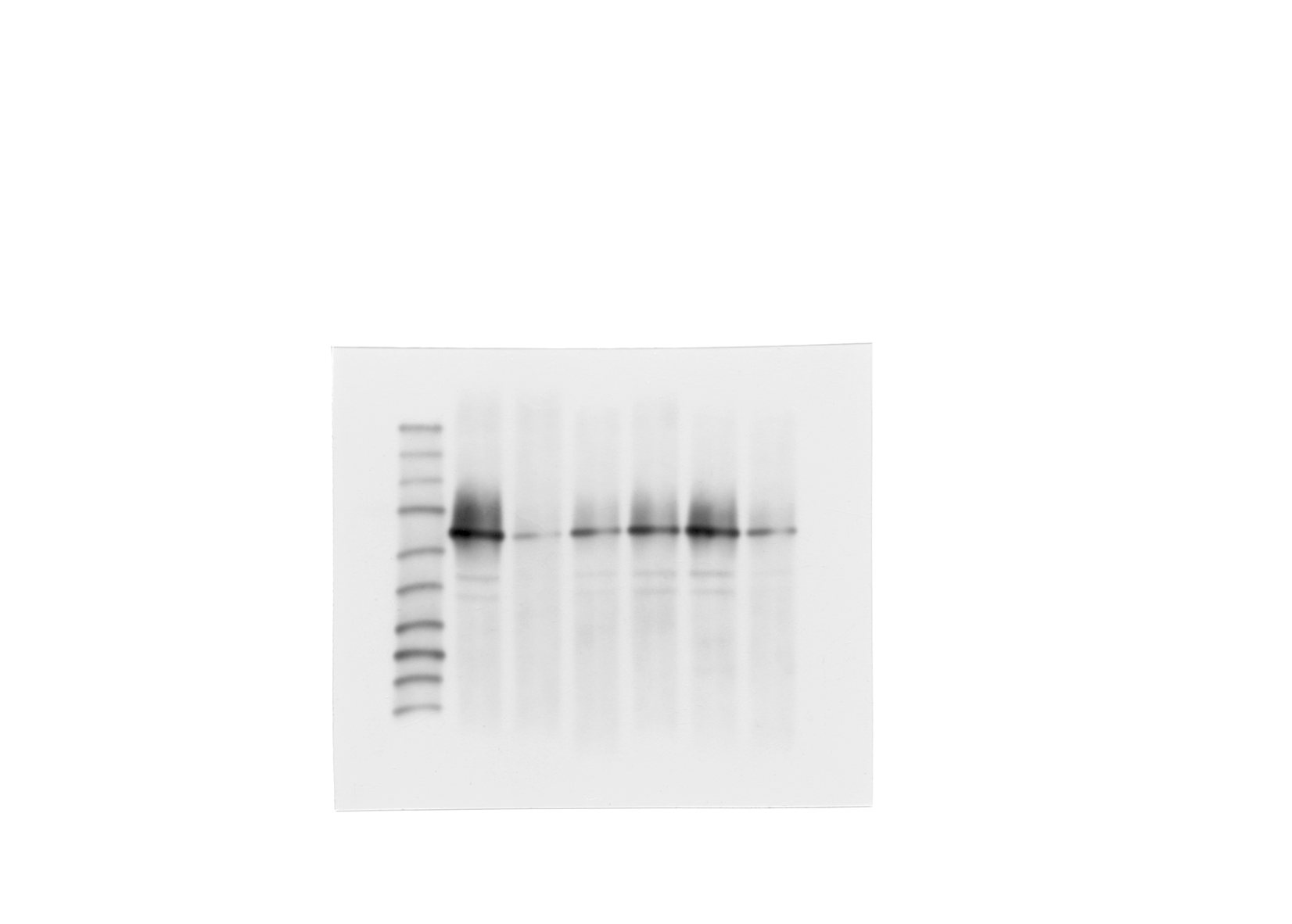

Supplement: Supplementary file 1 [file DataSheet1.zip › Image of the original Western blots/Nrf2 68 kDa 2.tif]

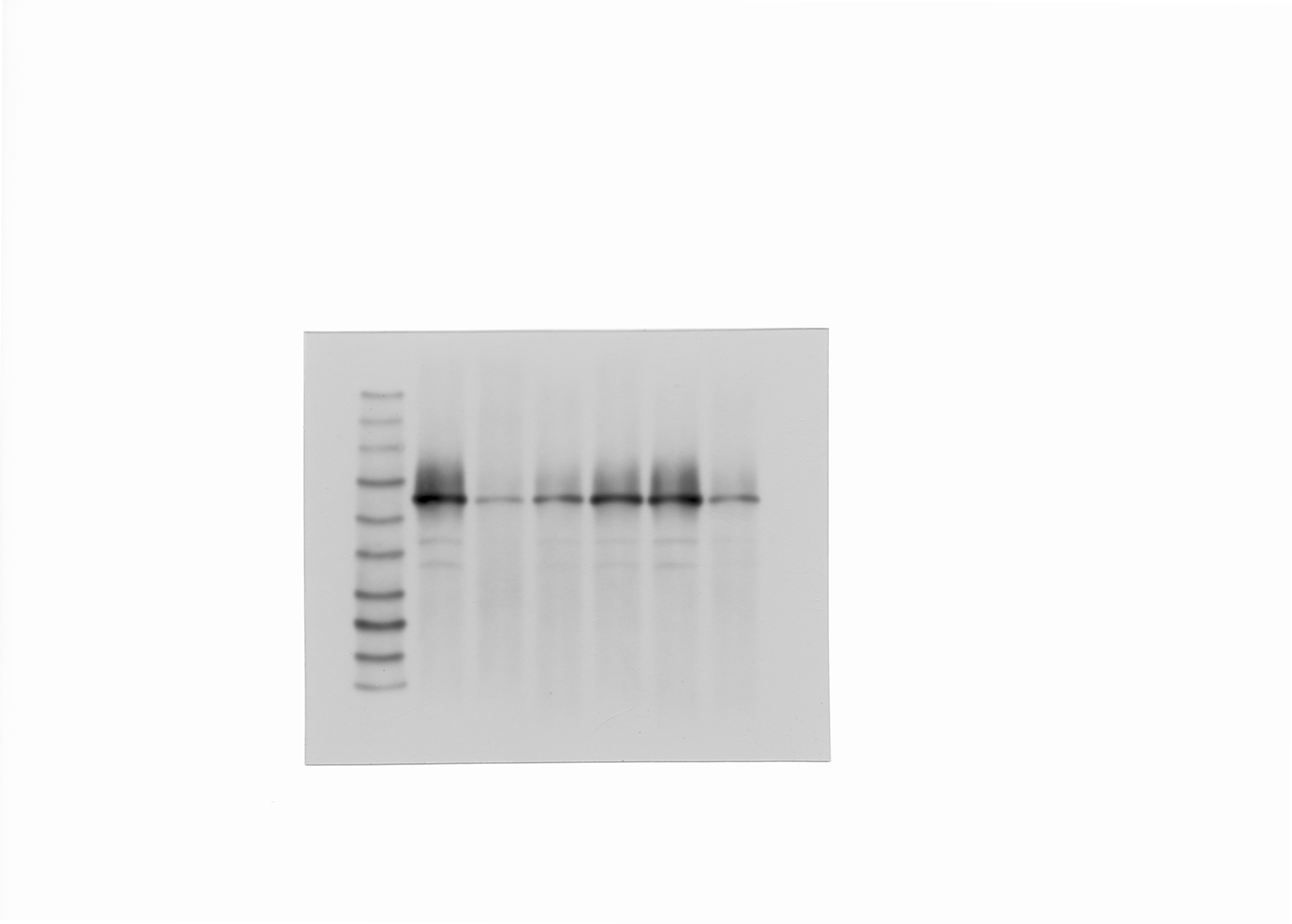

Supplement: Supplementary file 1 [file DataSheet1.zip › Image of the original Western blots/Nrf2 68 kDa 3.tif]

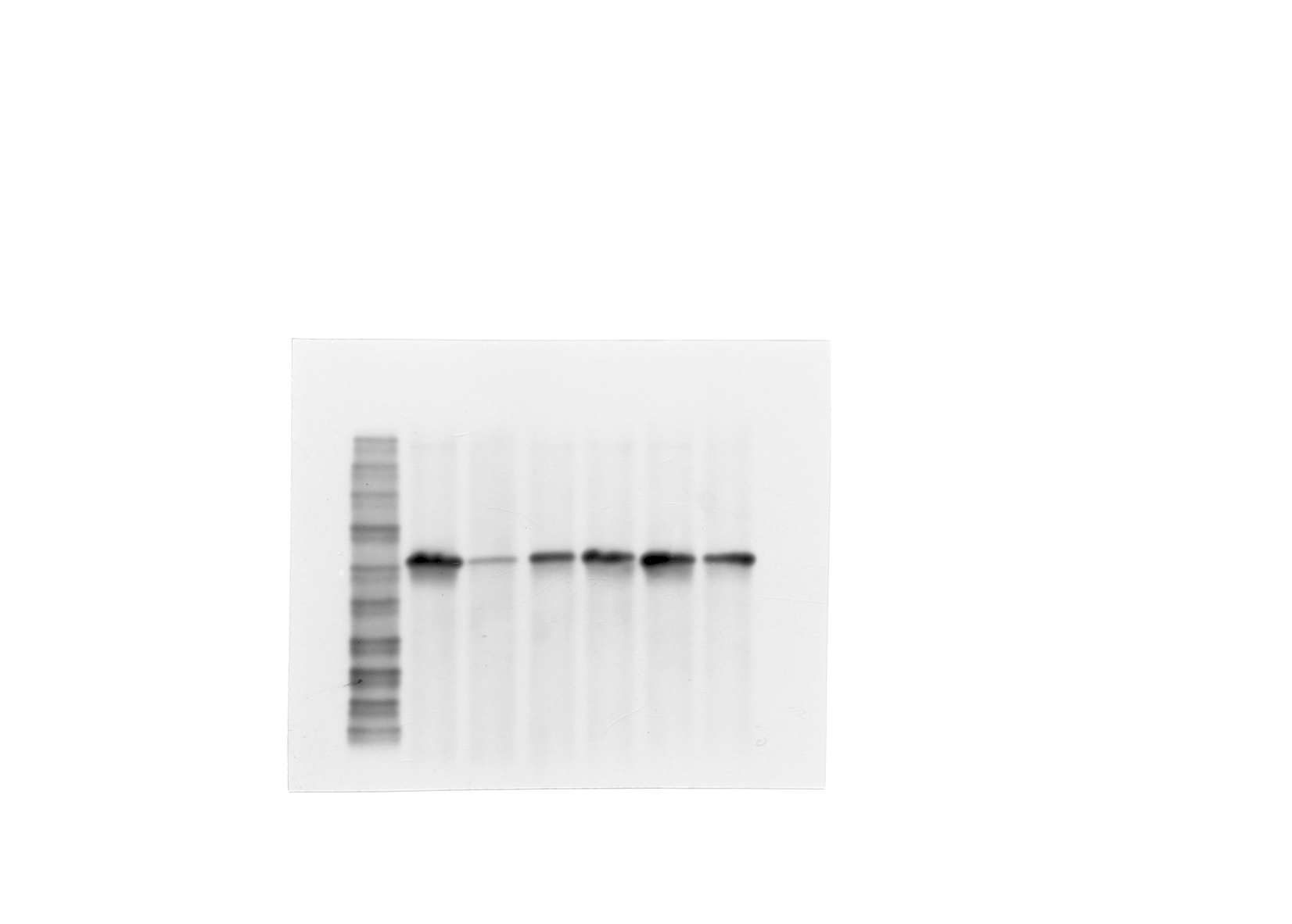

Supplement: Supplementary file 1 [file DataSheet1.zip › Image of the original Western blots/Occludin 62 kDa 1.tif]

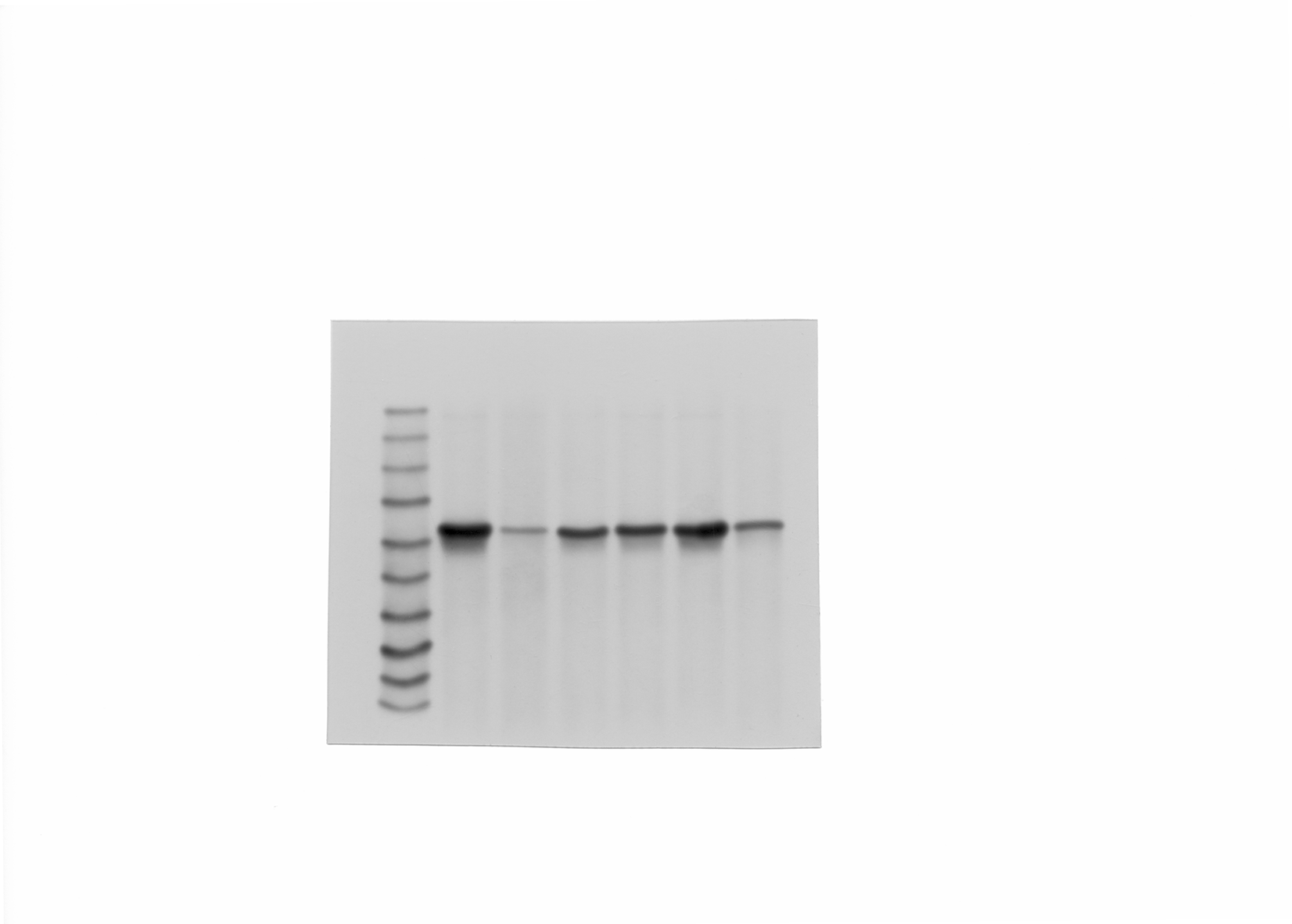

Supplement: Supplementary file 1 [file DataSheet1.zip › Image of the original Western blots/Occludin 62 kDa 2.tif]

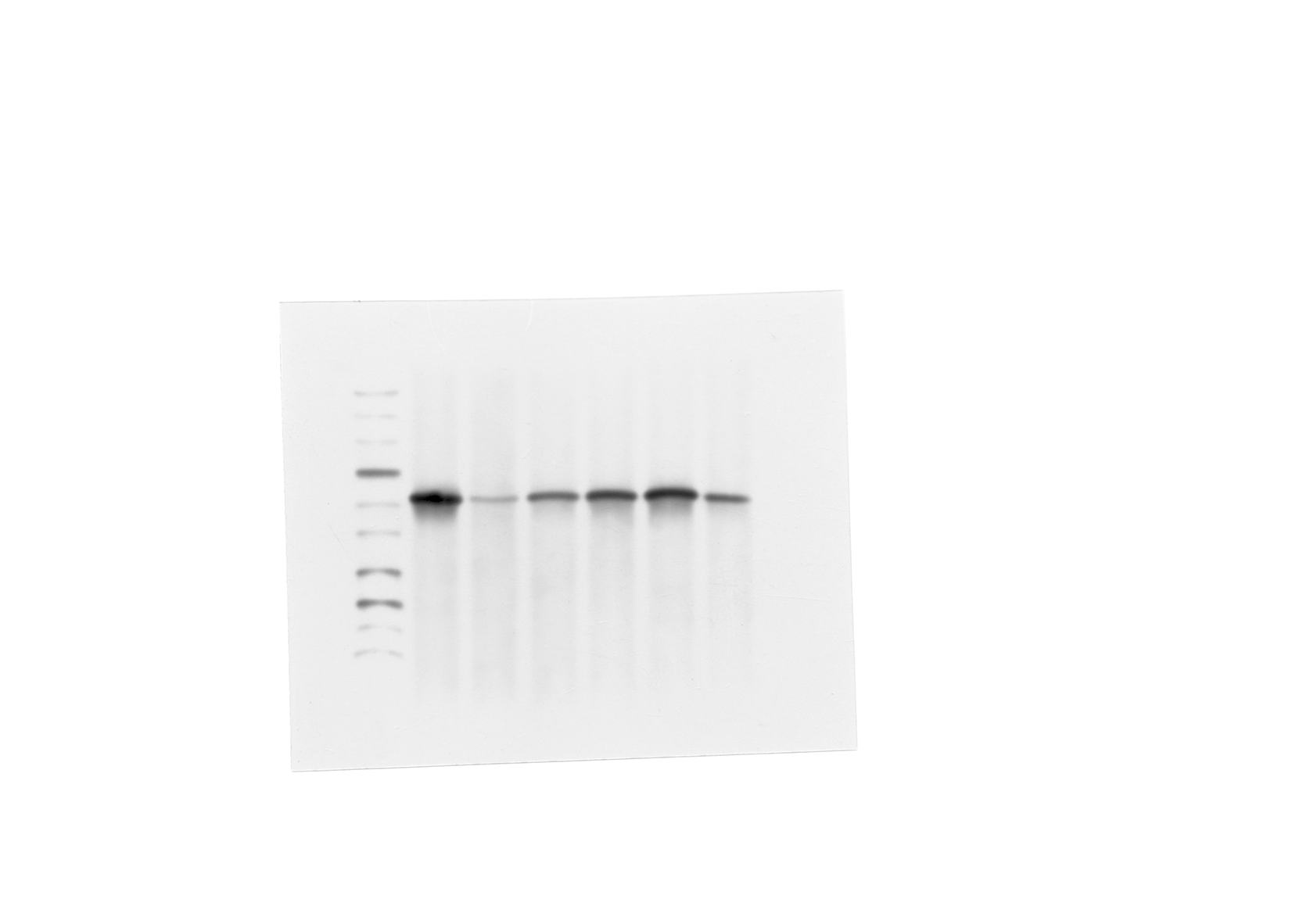

Supplement: Supplementary file 1 [file DataSheet1.zip › Image of the original Western blots/Occludin 62 kDa 3.tif]

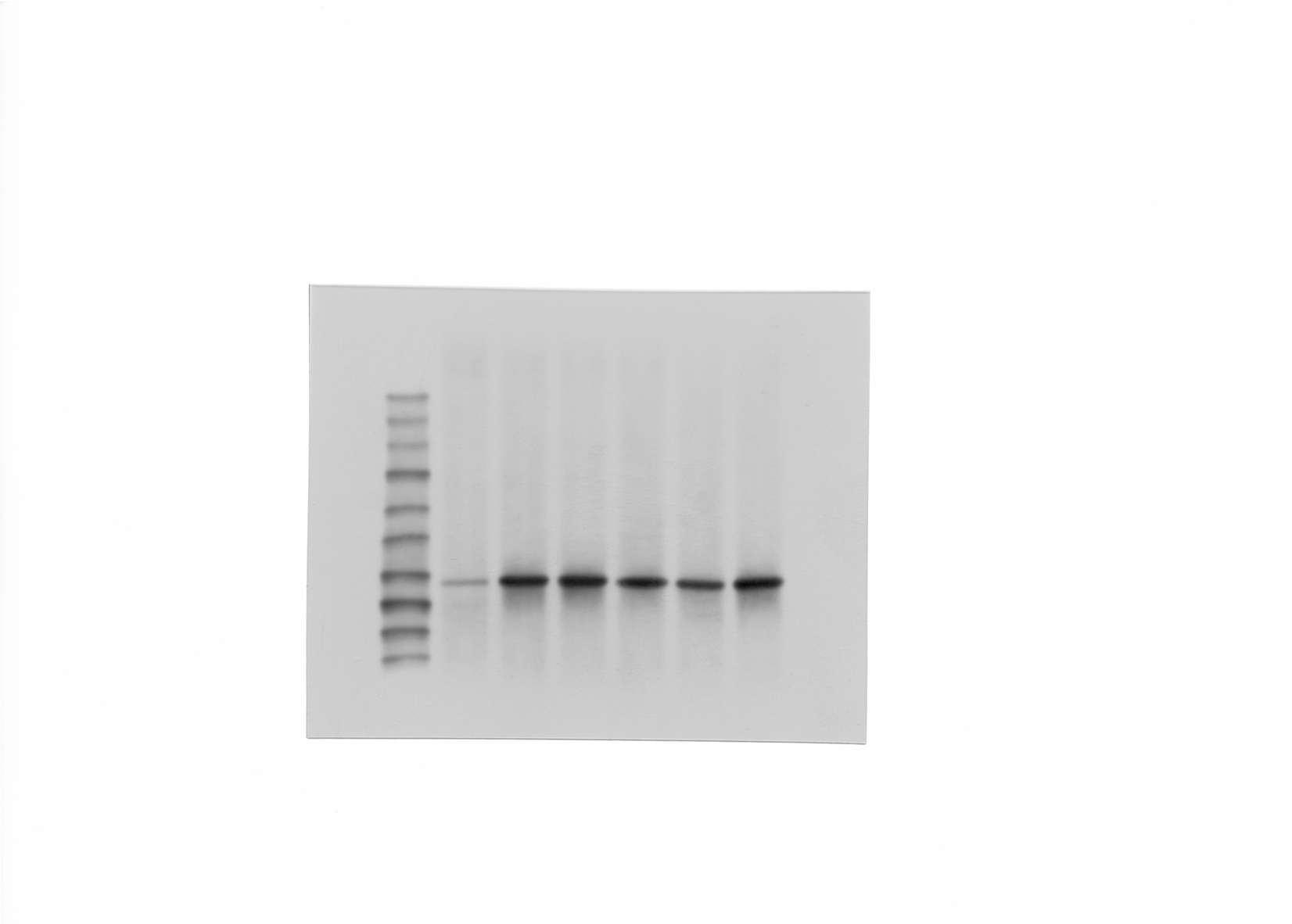

Supplement: Supplementary file 1 [file DataSheet1.zip › Image of the original Western blots/p-Ia╩Ba┴ 39 kDa 1.tif]

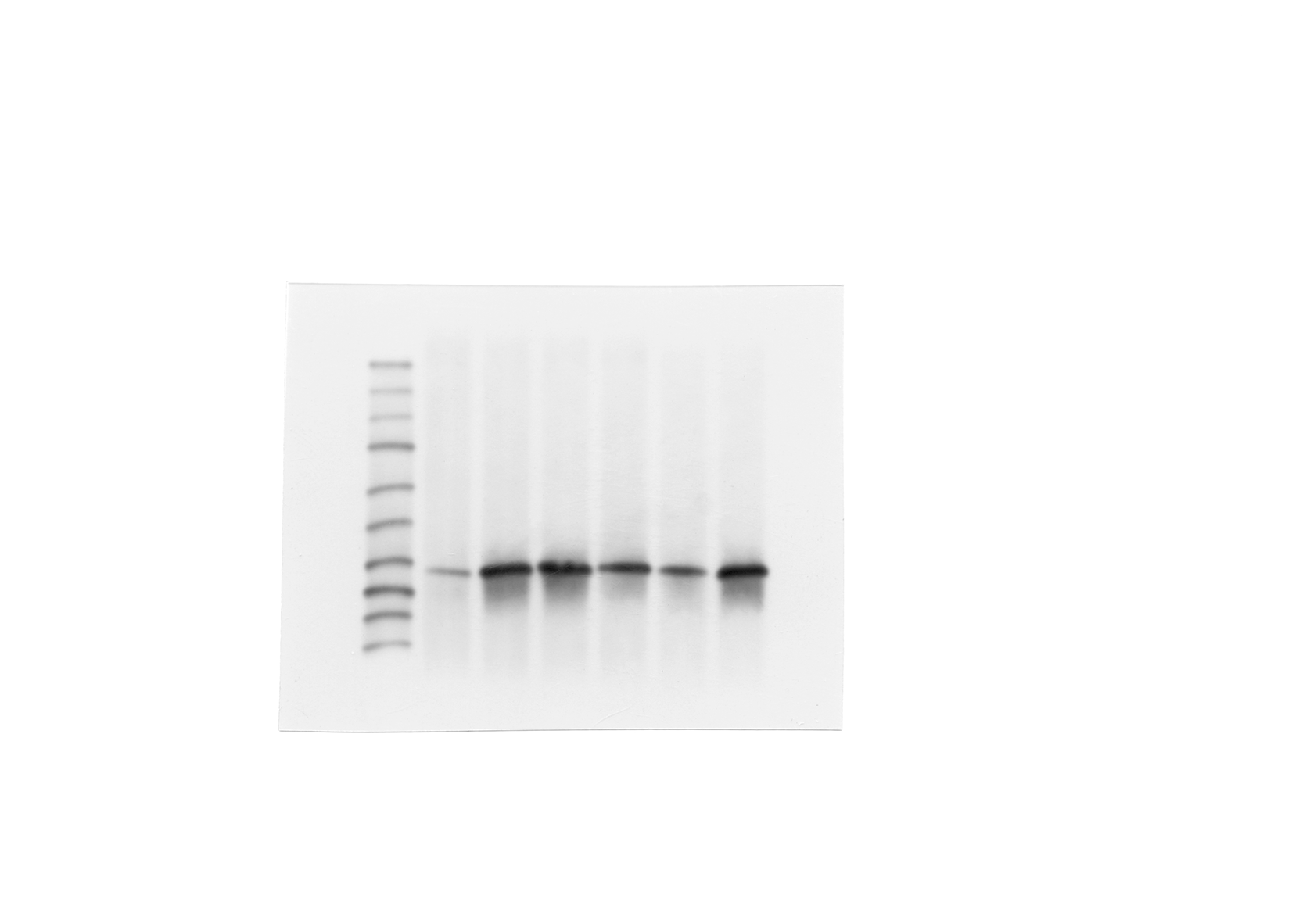

Supplement: Supplementary file 1 [file DataSheet1.zip › Image of the original Western blots/p-Ia╩Ba┴ 39 kDa 2.tif]

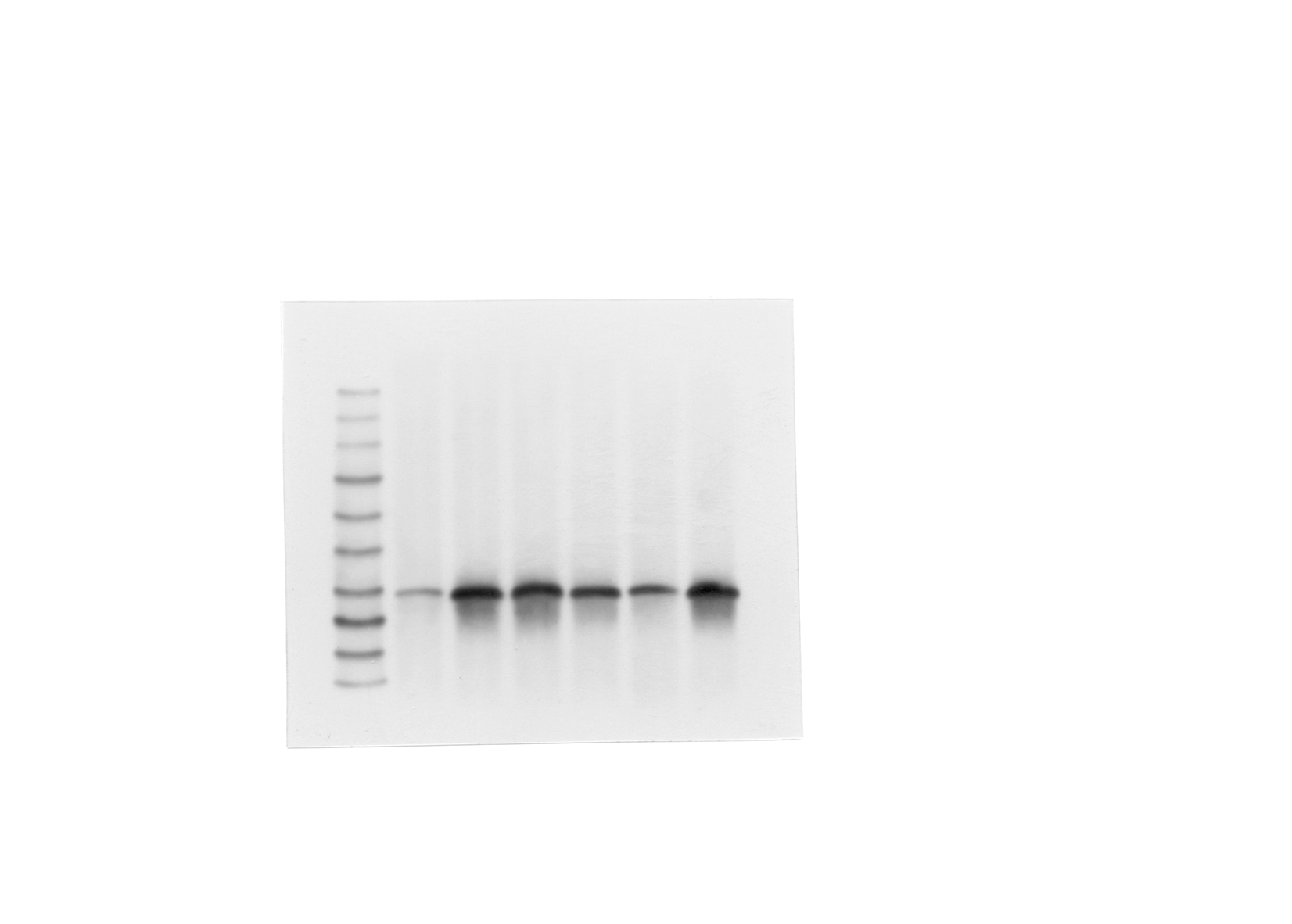

Supplement: Supplementary file 1 [file DataSheet1.zip › Image of the original Western blots/p-Ia╩Ba┴ 39 kDa 3.tif]

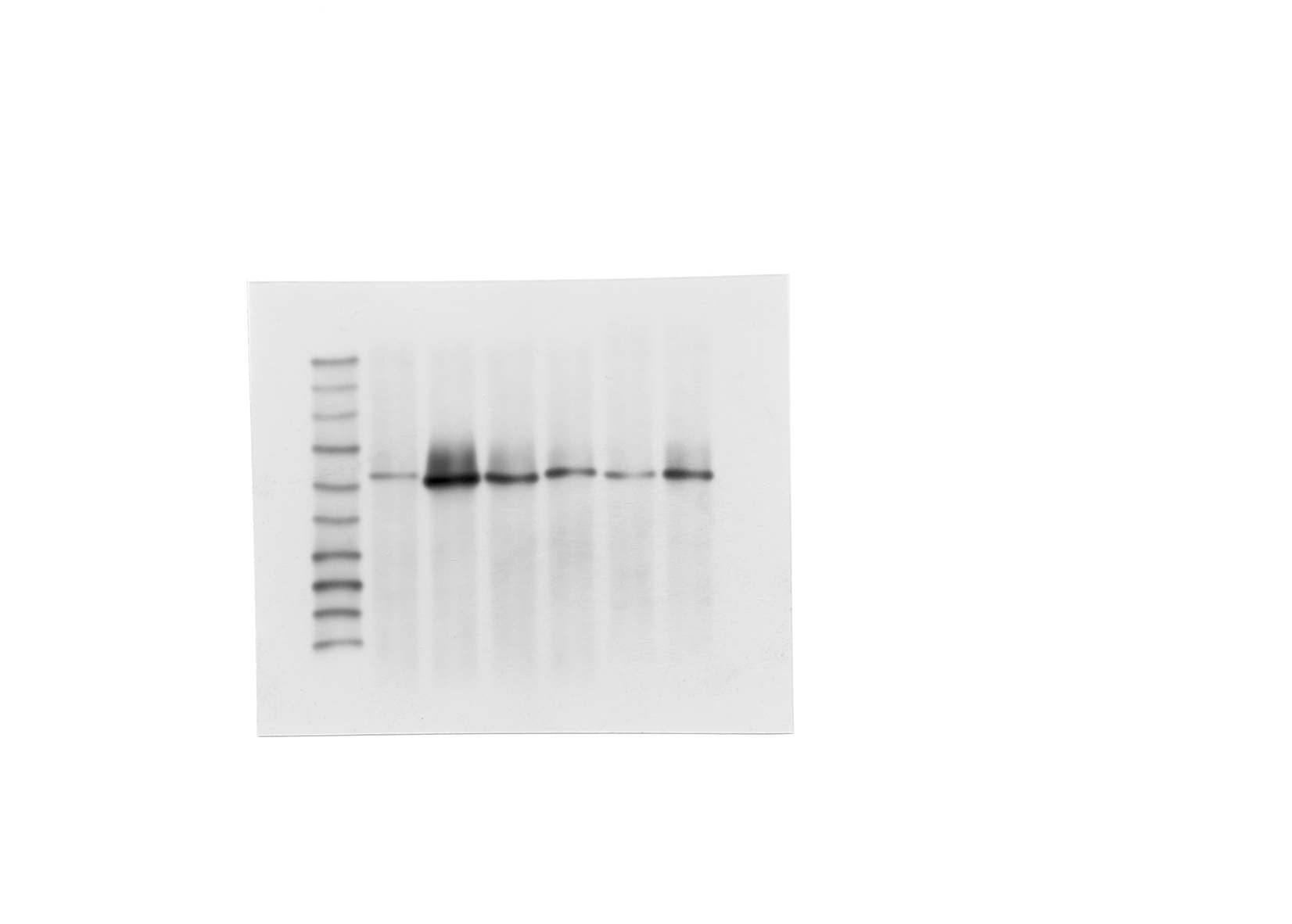

Supplement: Supplementary file 1 [file DataSheet1.zip › Image of the original Western blots/p65(nuclear) 65 kDa 1.tif]

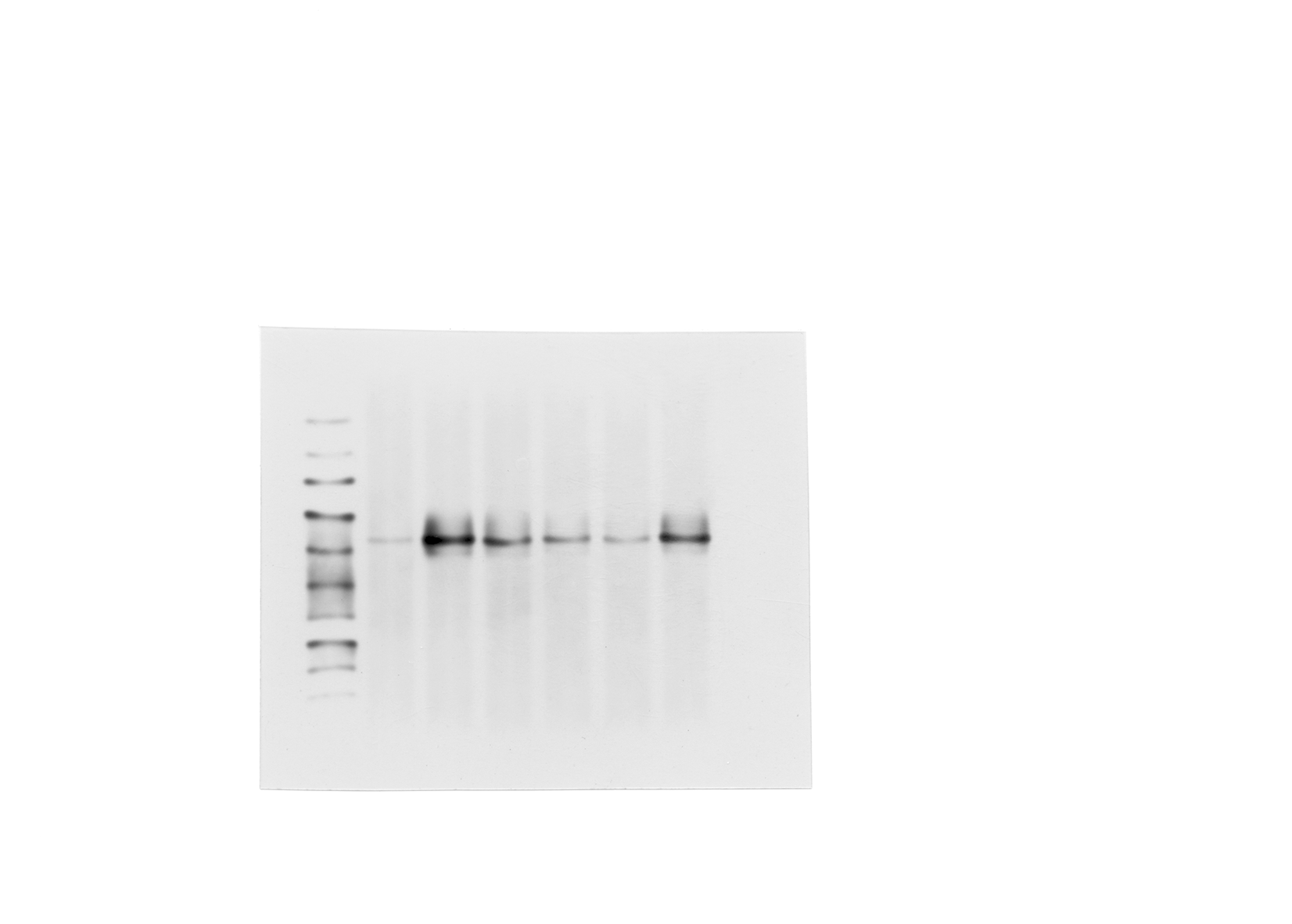

Supplement: Supplementary file 1 [file DataSheet1.zip › Image of the original Western blots/p65(nuclear) 65 kDa 2.tif]

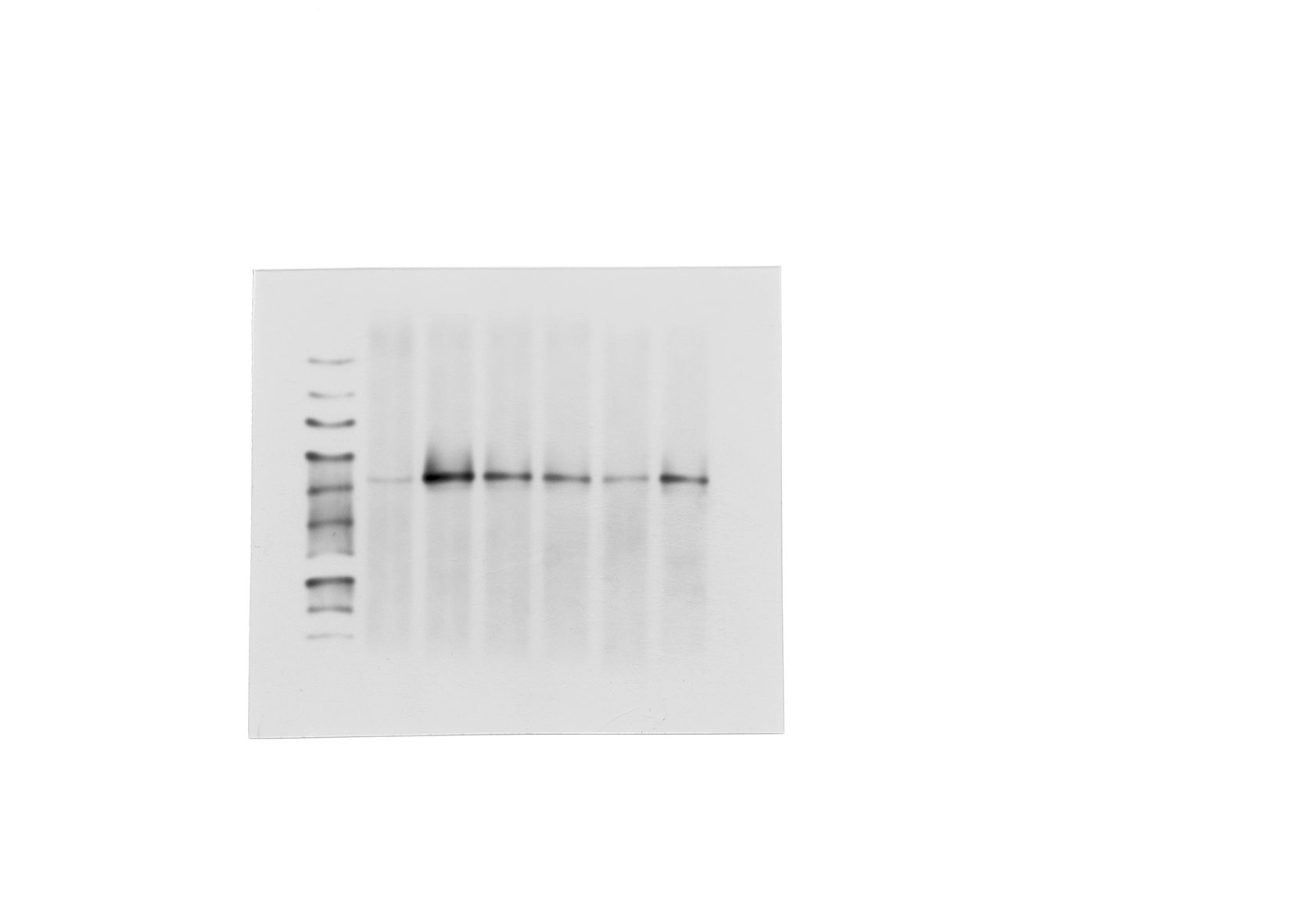

Supplement: Supplementary file 1 [file DataSheet1.zip › Image of the original Western blots/p65(nuclear) 65 kDa 3.tif]

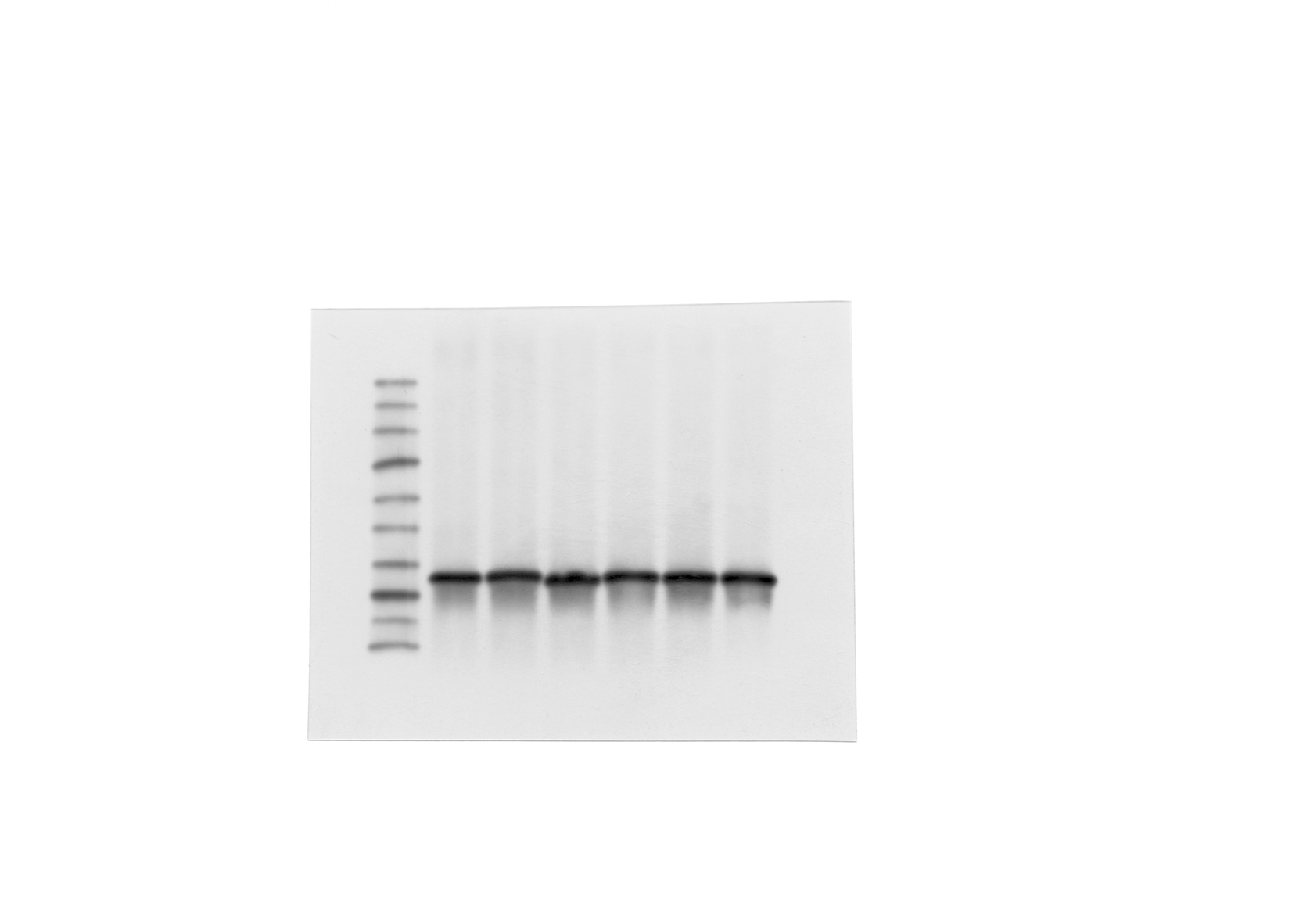

Supplement: Supplementary file 1 [file DataSheet1.zip › Image of the original Western blots/PCNA 36 kDa 1.tif]

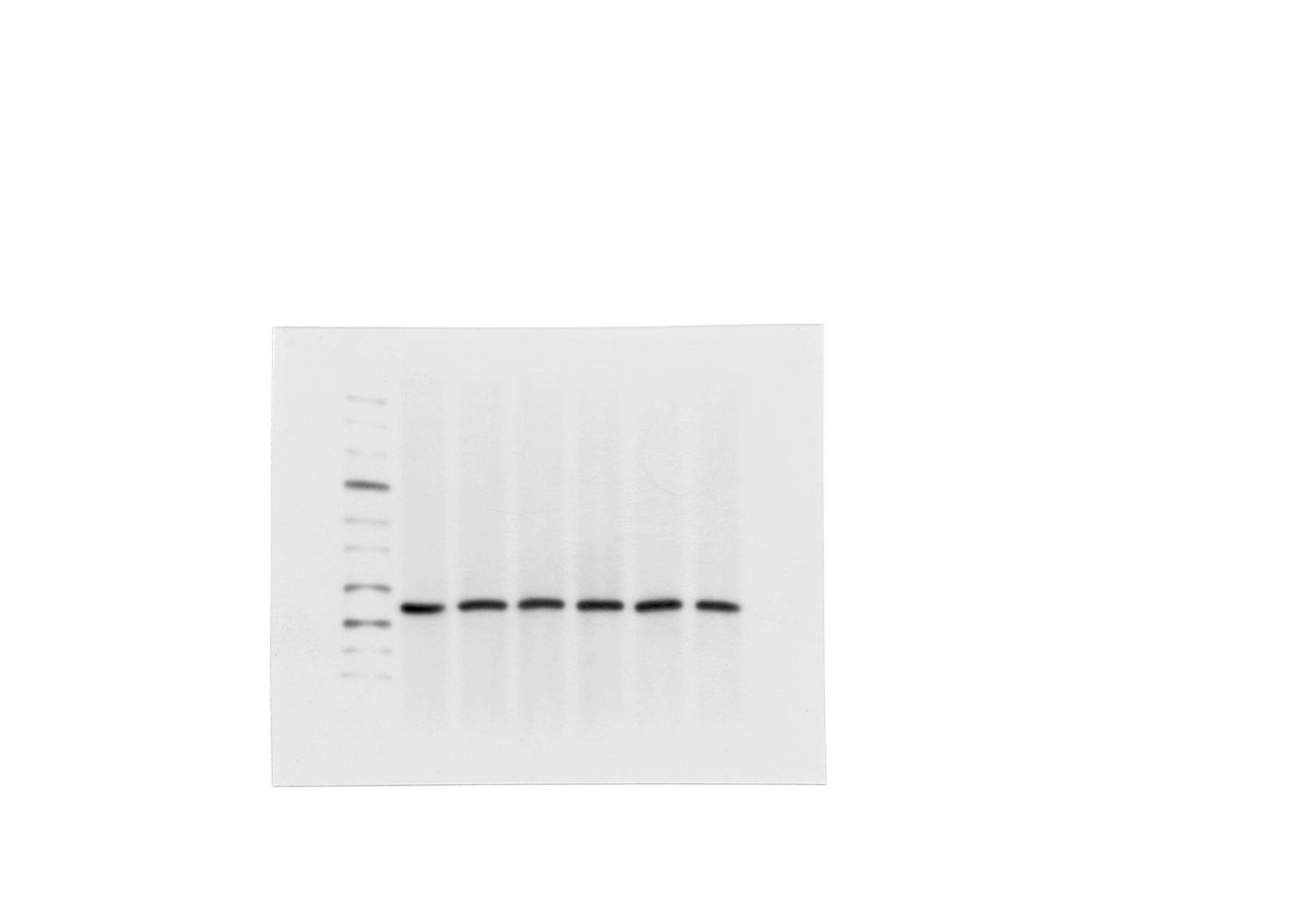

Supplement: Supplementary file 1 [file DataSheet1.zip › Image of the original Western blots/PCNA 36 kDa 2.tif]

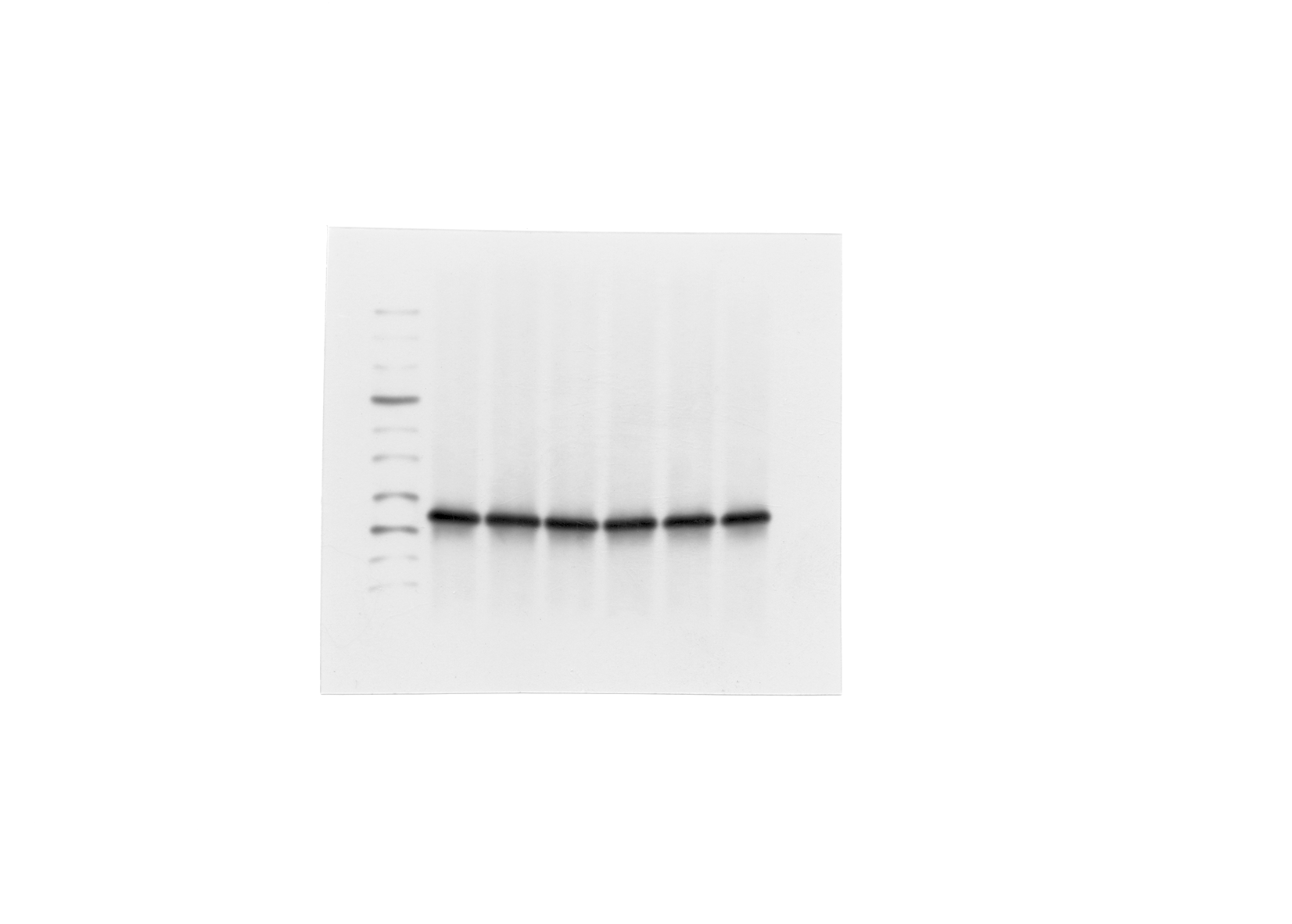

Supplement: Supplementary file 1 [file DataSheet1.zip › Image of the original Western blots/PCNA 36 kDa 3.tif]

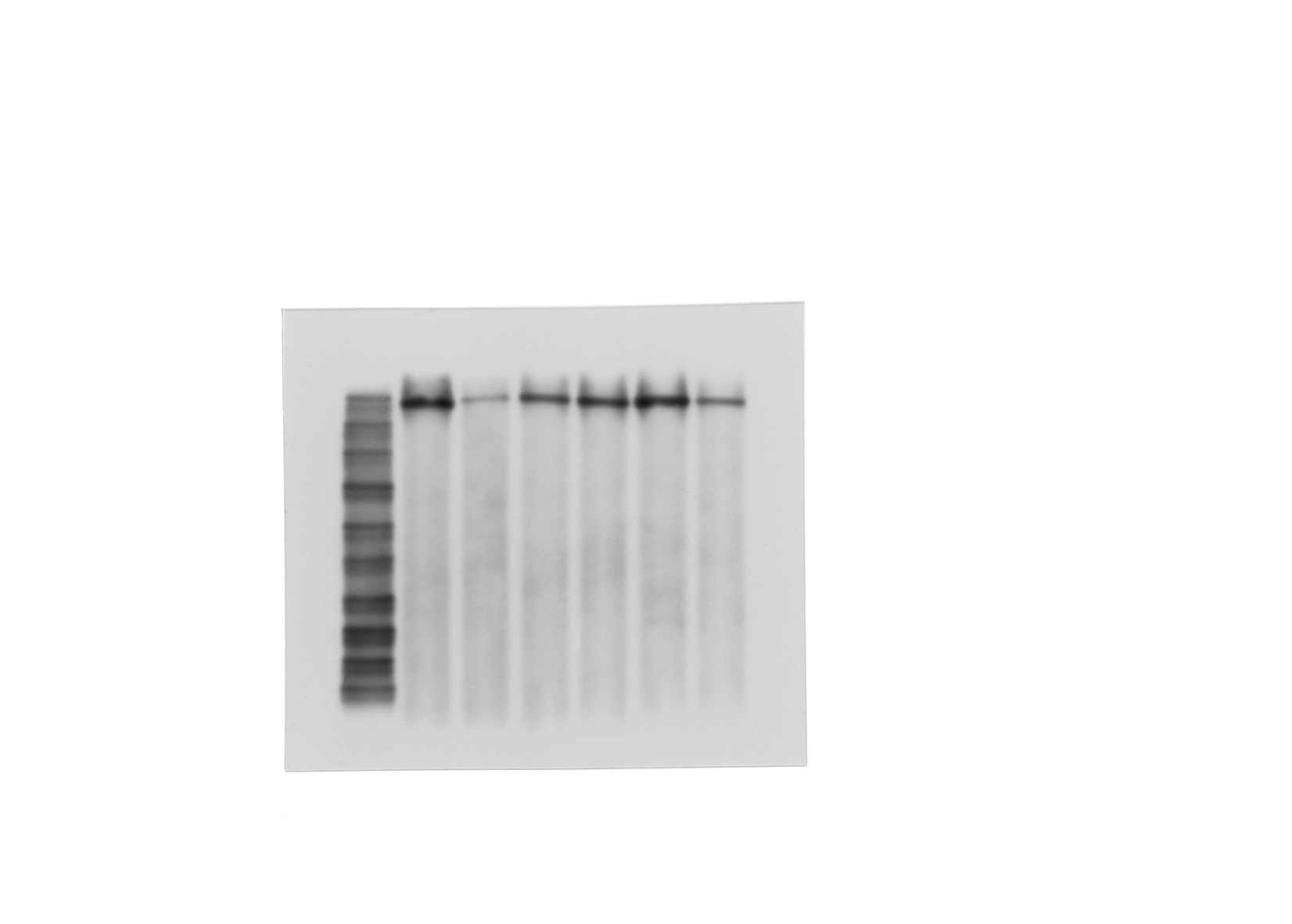

Supplement: Supplementary file 1 [file DataSheet1.zip › Image of the original Western blots/ZO-1 195 kDa 1.tif]

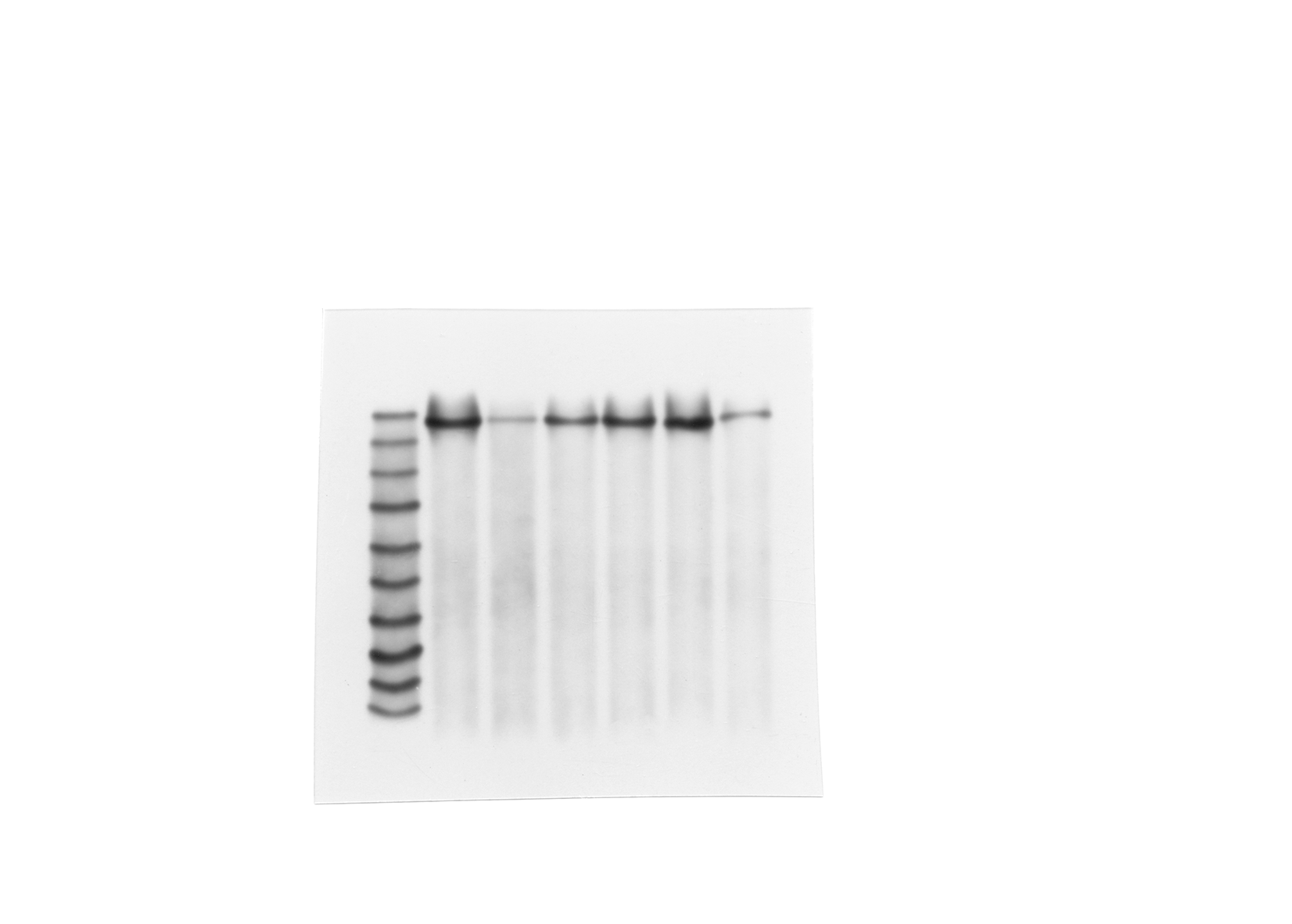

Supplement: Supplementary file 1 [file DataSheet1.zip › Image of the original Western blots/ZO-1 195 kDa 2.tif]

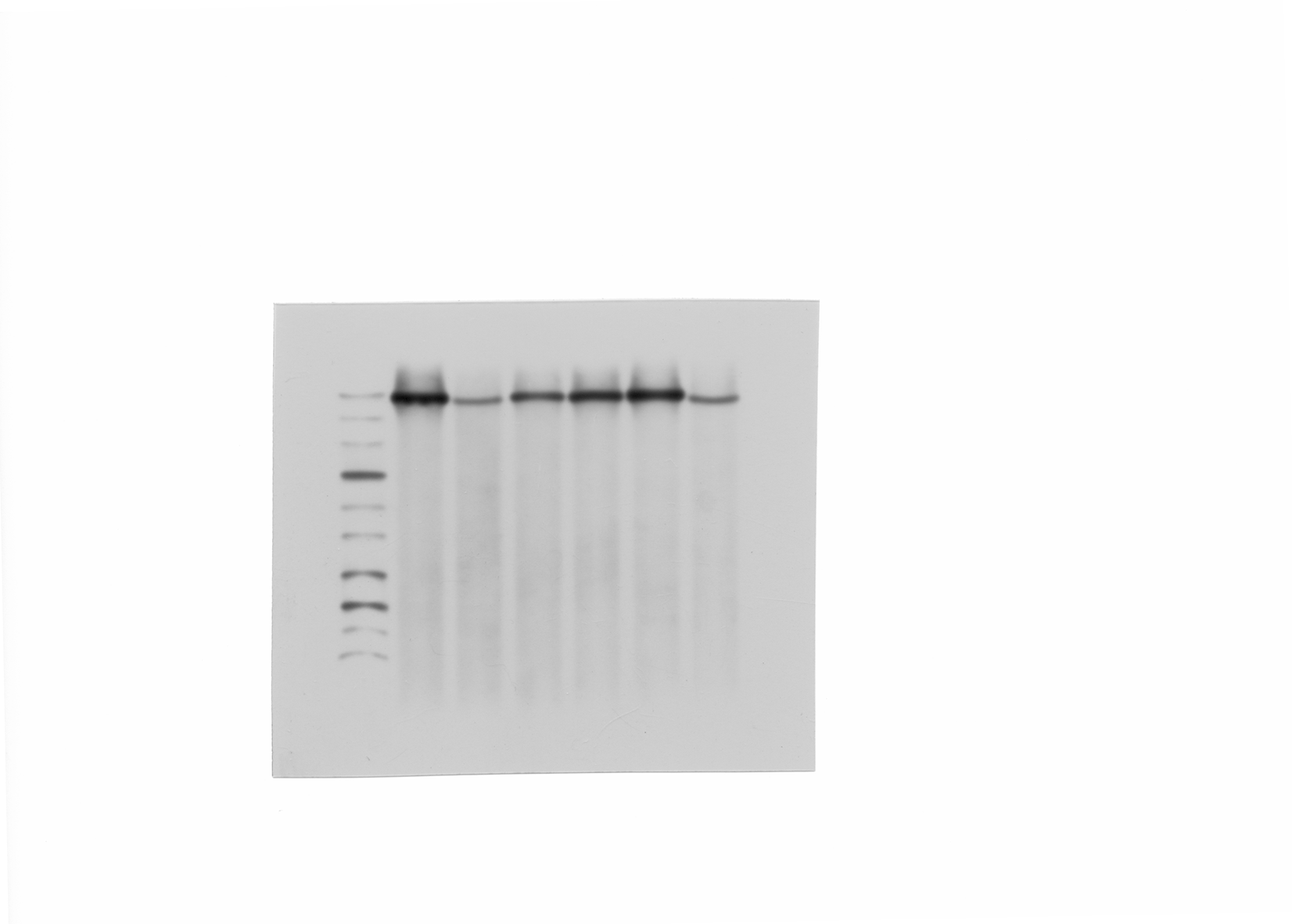

Supplement: Supplementary file 1 [file DataSheet1.zip › Image of the original Western blots/ZO-1 195 kDa 3.tif]

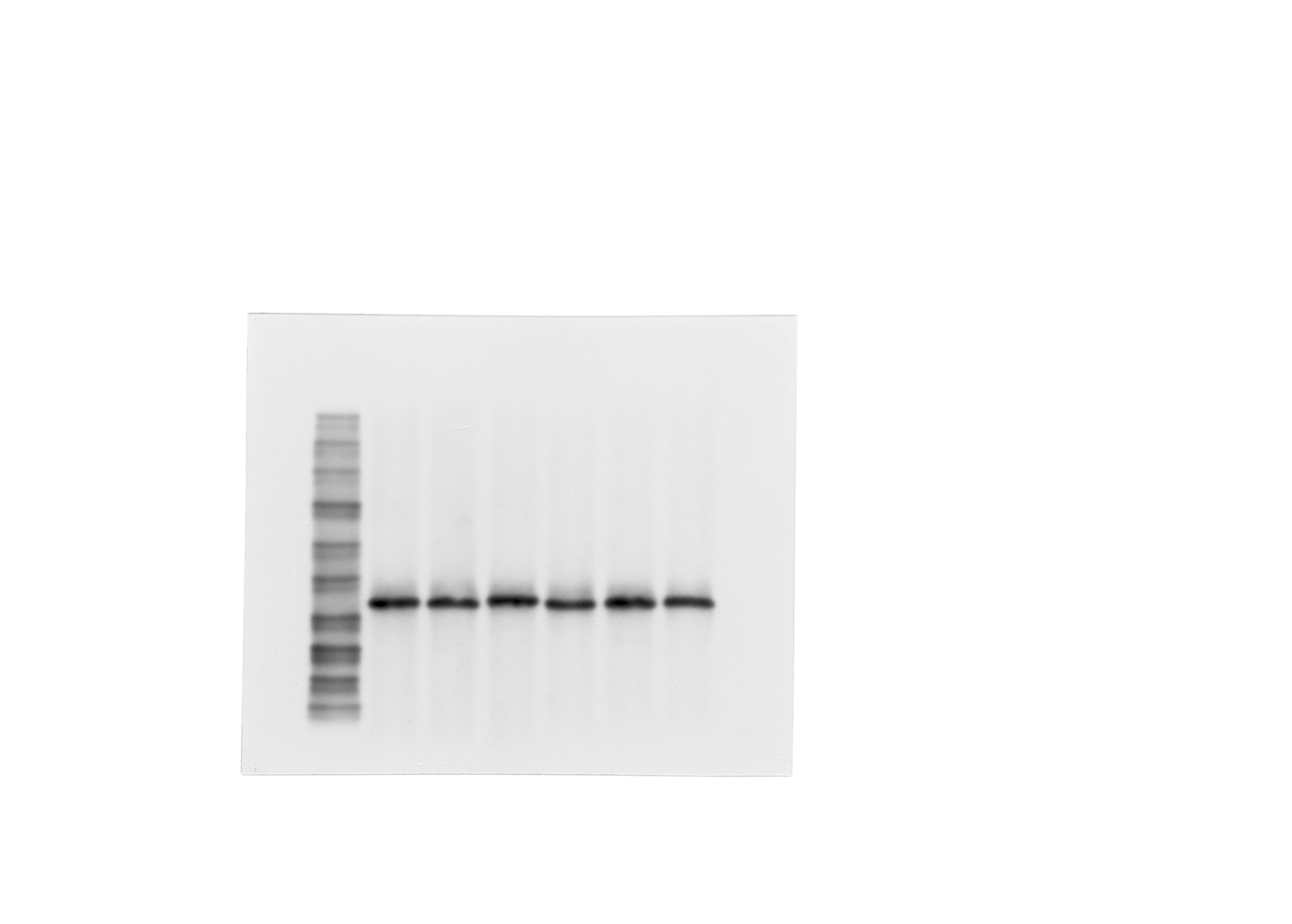

Supplement: Supplementary file 1 [file DataSheet1.zip › Image of the original Western blots/a┬-actin 43kDa 1.tif]

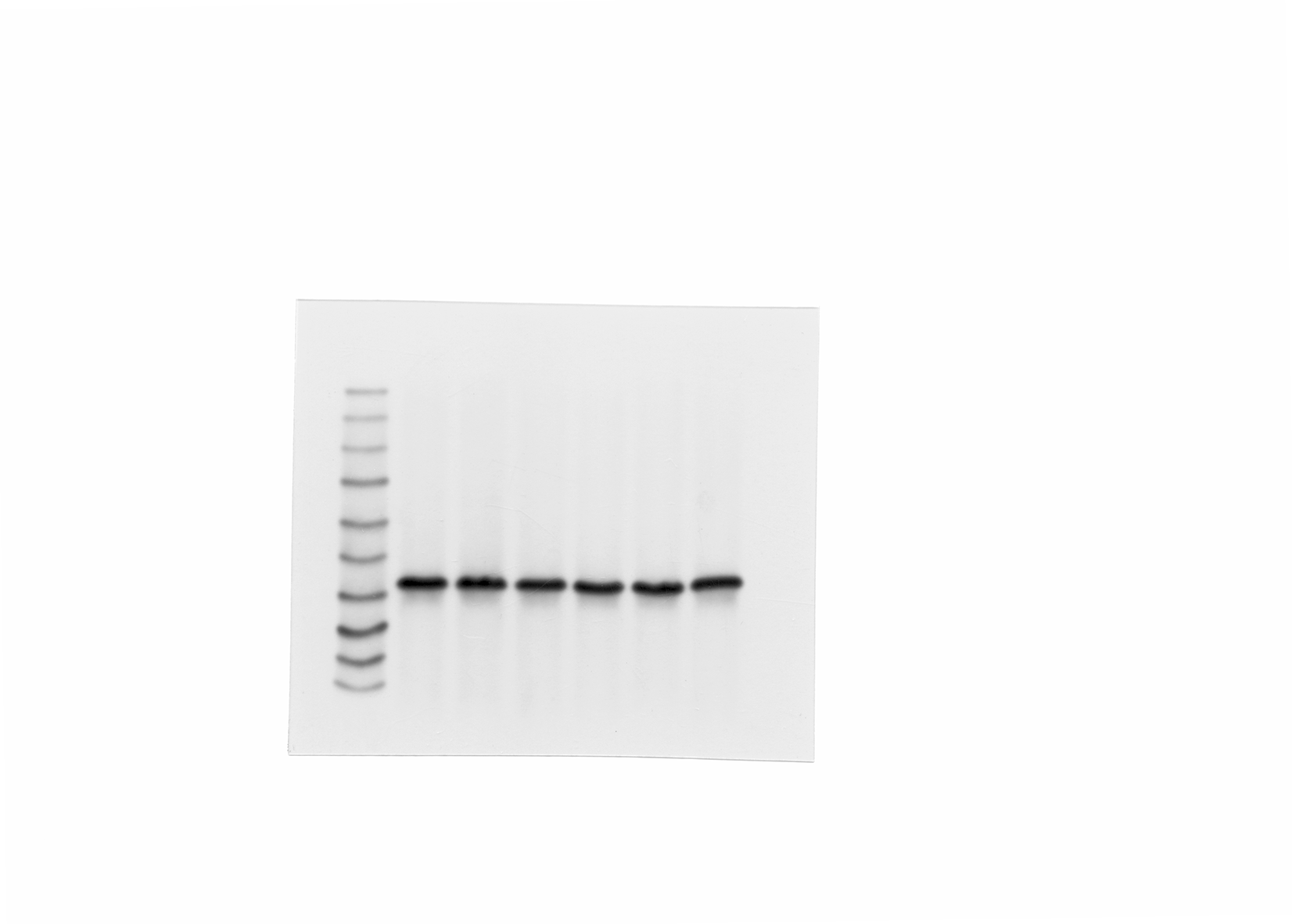

Supplement: Supplementary file 1 [file DataSheet1.zip › Image of the original Western blots/a┬-actin 43kDa 2.tif]

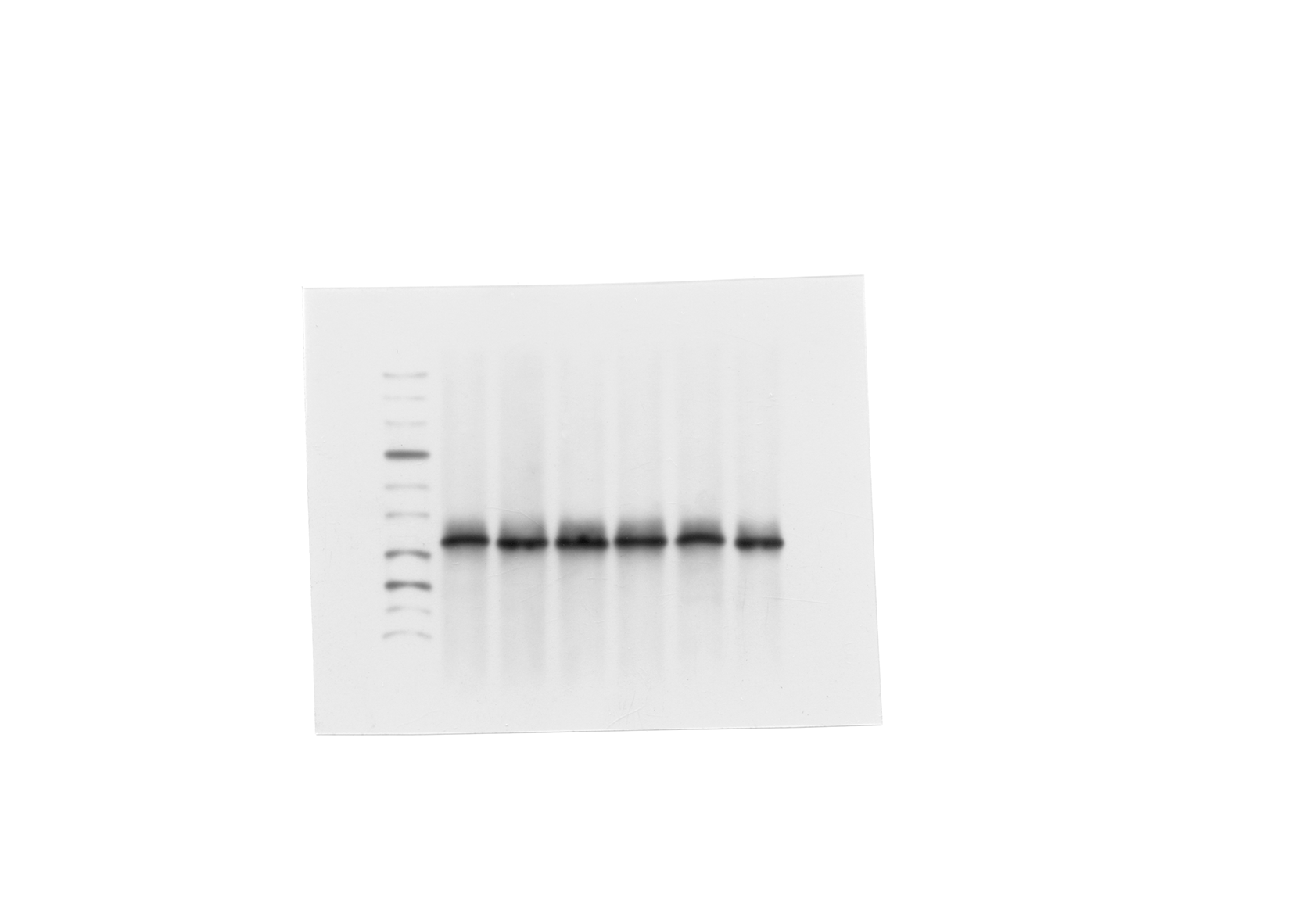

Supplement: Supplementary file 1 [file DataSheet1.zip › Image of the original Western blots/a┬-actin 43kDa 3.tif]
